# Anti-Inflammatory Activity of Black Soldier Fly Oil Associated with Modulation of TLR Signaling: A Metabolomic Approach

**DOI:** 10.3390/ijms241310634

**Published:** 2023-06-25

**Authors:** Hadas Richter, Ofer Gover, Betty Schwartz

**Affiliations:** Institute of Biochemistry, Food Science and Nutrition, The School of Nutritional Sciences, The Robert H. Smith Faculty of Agriculture, Food and Environment, The Hebrew University of Jerusalem, Rehovot 761001, Israel

**Keywords:** black soldier fly larvae (BSFL), medium chain fatty acid (MCFA) C12:0, dextran sulfate sodium (DSS)-induced colitis, macrophage, Toll-like receptor (TLR), lipopolysaccharides (LPS), Pam3CSK4, proinflammatory cytokine, mammalian target of rapamycin (mTOR), peroxisome proliferator-activated receptor (PPAR)

## Abstract

Dietary intervention in the treatment of ulcerative colitis involves, among other things, modifications in fatty acid content and/or profile. For example, replacing saturated long chain fatty acids with medium chain fatty acids (MCFAs) has been reported to ameliorate inflammation. The Black Soldier Fly Larvae’s (BSFL) oil is considered a sustainable dietary ingredient rich in the MCFA C12:0; however, its effect on inflammatory-related conditions has not been studied until now. Thus, the present study aimed to investigate the anti-inflammatory activity of BSFL oil in comparison to C12:0 using TLR4- or TLR2-activated THP-1 and J774A.1 cell lines and to assess its putative protective effect against dextran sulfate sodium (DSS)-induced acute colitis in mice. BSFL oil and C12:0 suppressed proinflammatory cytokines release in LPS-stimulated macrophages; however, only BSFL oil exerted anti-inflammatory activity in Pam3CSK4-stimulated macrophages. Transcriptome analysis provided insight into the possible role of BSFL oil in immunometabolism switch, involving mTOR signaling and an increase in PPAR target genes promoting fatty acid oxidation, exhibiting a discrepant mode of action compared to C12:0 treatment, which mainly affected cholesterol biosynthesis pathways. Additionally, we identified anti-inflammatory eicosanoids, oxylipins, and isoprenoids in the BSFL oil that may contribute to an orchestrated anti-inflammatory response. In vivo, a BSFL oil-enriched diet (20%) ameliorated the clinical signs of colitis, as indicated by improved body weight recovery, reduced colon shortening, reduced splenomegaly, and an earlier phase of secretory IgA response. These results indicate the novel beneficial use of BSFL oil as a modulator of inflammation.

## 1. Introduction

Multiple dietary approaches have been adopted to evaluate the effects of various fatty acids (FAs) on inflammatory bowel disease (IBD) and one of its main forms, ulcerative colitis (UC). Toll-like receptors (TLRs) are pattern-recognition receptors (PRRs), key components of the host immune system, which facilitate the recognition of pathogen-associated molecular patterns (PAMPS) and initiate inflammatory signaling to effectively clear the insult However, the excessive stimulation of TLRs can cause an imbalance between the proinflammatory and anti-inflammatory factors that underlie the clinical features found in inflammatory diseases. The expression of the genes coding for TLR 2, 4, 8, and 9 was reported to be upregulated in patients with active UC [1]. Typically, the activation of TLR4 is preceded by the binding of Gram-negative bacterial lipopolysaccharides (LPS) to the CD14 protein, which is then transferred to a TLR4/myeloid differentiation protein 2 (MD2) complex to form a heterotetramer [2]. The lipid A region of LPS contains numerous saturated fatty acyl chains, including lauric acid (C12:0) [3], that are required for binding to the hydrophobic pocket of MD2 [4,5]. TLR2/TLR1 and TLR2/TLR6 heterodimers are stimulated by tri-acylated and di-acylated lipopeptides, respectively [6,7]. TLRs activation may lead to several possible signaling cascades. The engagement of the myeloid differentiation primary response 88 (MyD88) and/or the Toll/IL-1R domain-containing adaptor-inducing IFN-β (TRIF) adaptor proteins ultimately leads to the activation of transcription factors such as the nuclear factor-kappa B (NF-κB), activator protein-1 (AP-1), and interferon regulatory factor 3 (IRF3), resulting in an increase in the gene transcription of proinflammatory factors [2,8]. The phosphoinositide 3-kinase (PI3K)/Akt pathway, which may also be activated upon TLR dimerization, is reported to be involved in immunometabolism, regulating macrophage polarization phenotypes through the mammalian target of rapamycin (mTOR) complex 1 [9]. The two extremes of functional polarization phenotypes are defined as M1 (classically activated) and M2 (alternatively activated) macrophages, and they use distinct metabolic programs to fuel their functions. Whereas M1-like macrophages are reportedly driven via mTORC1 signaling, leading to the expression of glycolytic genes and inflammatory cytokines, M2-like macrophages reportedly utilize fatty acid oxidation (FAO) while resolving inflammation [10,11].

Black soldier fly (*Hermetia illucens*) larvae (BSFL) are considered the new “superstar” producers of sustainable feed products since they provide high-quality protein. The protein fraction is already industrialized as a premium food ingredient, but the remaining oil extract goes mostly unexploited. However, BSFL oil has a unique profile comprising 40–50% lauric acid (C12:0), a saturated medium chain fatty acid (MCFA) carrying a backbone chain of 12 carbon atoms. C12:0 is known for its antimicrobial effects on Gram-positive bacteria, including gut bacteria [12]. In addition, MCFAs have distinct immunomodulatory properties. Diets enriched with MCFAs were reported to suppress inflammation in experimental models of dextran sulfate sodium (DSS)-induced colitis compared to diets supplemented with long chain fatty acids [13], lard [14], or ω-6 enriched formulas [15]. The inhibitory cross-talk between C12:0 and TLR/NF-κB signaling was evidenced in vivo in LPS-induced liver inflammation in rats [16] and in *P. acnes*-induced ear edema in mice [17]. Additionally, MCFAs were also demonstrated to enhance the expression of secretory immunoglobulin A (IgA) in response to LPS in the rat intestine [18]. Secreted IgA plays a critical role in the defense against pathogens and has been reported to induce a therapeutic effect on colitis models in mice [19]. In addition to C12:0, BSFL oil contains monounsaturated palmitoleic (C16:1) and oleic (C18:1) acids. These FAs have been previously reported to increase the gut microbiota diversity of healthy and unhealthy animal models, resulting in diminished symptoms of IBD [20]. 

TLRs antagonists have been suggested to exert potential therapeutic effects aimed to avoid intestinal inflammation [21,22]. In this context, nutritional therapy involving the development of hit or lead compounds able to modulate TLR signaling may be of interest in the management of inflammatory-related diseases. 

This study aimed to examine the anti-inflammatory activity of BSFL oil, explore a possible mechanism of action associated with TLR signaling in vitro, and provide a novel assessment of BSFL oil’s putative protective effects against DSS-induced colitis in vivo. 

## 2. Results

### 2.1. Suppression of TLR4-Mediated Proinflammatory Cytokines by Modified BSFL Oil (MBSFL) and C12:0 in Macrophages

The possible anti-inflammatory activity of BSFL oil was measured using in vitro systems of phorbol 12-myristate 13-acetate (PMA)-primed human THP-1 monocytes and murine J774A.1 macrophages stimulated with various TLR ligands. For these in vitro studies, the BSFL oil was saponified in order to form a modified BSFL oil (MBSFL) that was soluble in an aqueous environment. The FA C12:0, which comprises 42% of the BSFL oil’s FA composition (Supplementary Table S1) and is thought to play a role in mediating anti-inflammatory responses, was used as a reference for our in vitro studies. Consequently, for additional comparative treatments, we selected a natural pure lauric acid (LA) and a synthetic pure sodium laurate (SL).

**Figure 1:**
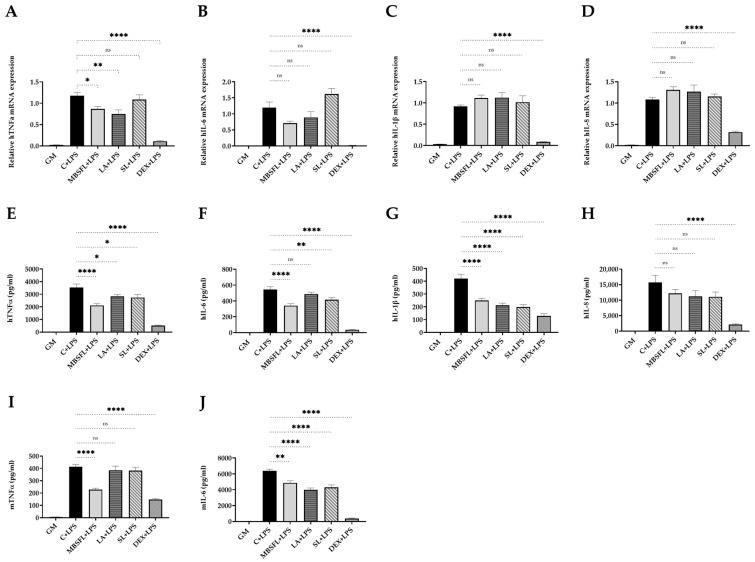
Suppression of Toll-like receptor (TLR) 4-mediated proinflammatory cytokines by modified BSFL oil (MBSFL) and C12:0 in macrophages. Phorbol 12-myristate 13-acetate (PMA)-primed human THP-1 and murine J774A.1 macrophages were treated with 250 µM MBSFL, 250 µM lauric acid (LA), 250 µM sodium laurate (SL), or dexamethasone (DEX; Positive control, 2 µg/mL in THP-1, 1.2 µg/mL in J774A.1), followed by lipopolysaccharides (LPS) stimulation, compared to stimulated vehicle-treated control (C + LPS), and untreated and unstimulated cells (GM; Negative control). (**A**–**D**) mRNA expression and; (**E**–**H**) Protein secretion levels of tumor necrosis factor alpha (TNFα), interleukin (IL)-6, IL-1β, and IL-8 in PMA-primed THP-1 cells stimulated with 10 ng/mL LPS for 12 h and 20 h, respectively; (**I**,**J**) TNFα and IL-6 levels in the medium of J774A.1 cells 24 h following 7.5 ng/mL LPS stimulation. mRNA expression was measured by qPCR (n = 4–6), and secreted cytokines in the medium were measured by ELISA (n = 8–10); ns: not significant, * *p* < 0.05, ** *p* < 0.01, **** *p* < 0.0001.

Even though that cell viability remained high (Supplementary Figure S2), MBSFL significantly reduced tumor necrosis factor alpha (TNFα) mRNA expression and protein secretion levels, both in LPS-stimulated THP-1 and in J774A.1 macrophages. LA and SL exhibited similar but less substantial effects, with a significant decrease in TNFα secretion only in THP-1 cells (Figure 1A,E,I). In addition, MBSFL, LA, and SL significantly reduced interleukin (IL)-6 and IL-1β protein secretion (Figure 1F,G,J) but had no significant effect on the respective cytokine transcription levels (Figure 1B,C, respectively). IL-8 mRNA and protein secretion levels were unaffected by MBSFL, LA, or SL treatment in LPS-stimulated THP-1 cells (Figure 1D,H). J774A.1 cells did not secrete IL-1β nor IL-8, neither in resting cells nor when cells were stimulated with LPS for 12 or 24 h.

### 2.2. MBSFL and C12:0 Reciprocally Modulate TLR2-Mediated Proinflammatory Cytokines Expression and Secretion in Macrophages

**Figure 2:**
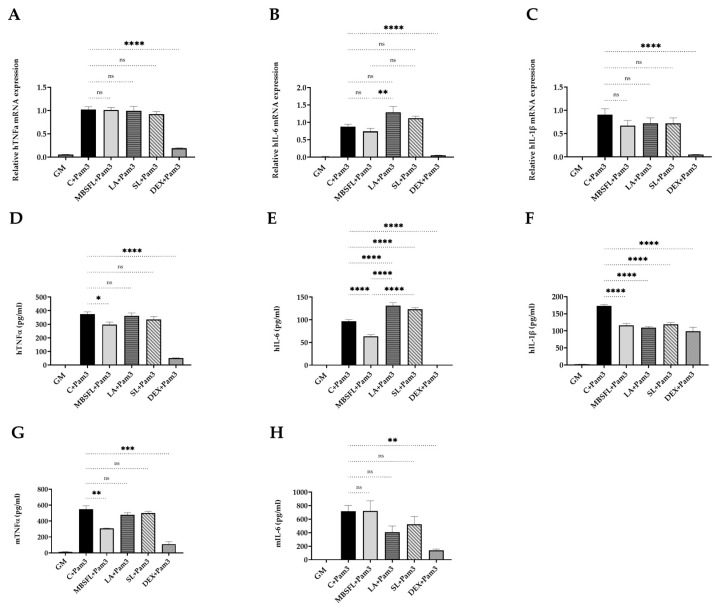

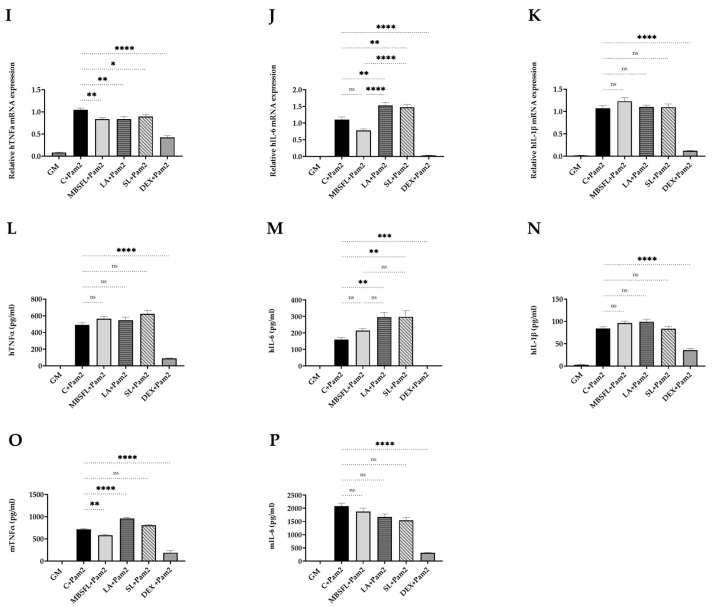
MBSFL and C12:0 reciprocally modulate TLR2-mediated proinflammatory cytokines expression and secretion in macrophages. PMA-primed THP-1 and J774A.1 macrophages were treated with 250 µM MBSFL, 250 µM LA, 250 µM SL, or DEX (positive control, 2 µg/mL in THP-1, 1.2 µg/mL in J774A.1), followed by stimulation with a TLR2 ligand and compared with stimulated vehicle-treated controls (C + Pam3 or C + Pam2) and untreated and unstimulated cells (GM; Negative control). (**A**–**C**) mRNA expression and; (**D**–**F**) Protein secretion levels of TNFα, IL-6, and IL-1β in THP-1 cells stimulated with 50 ng/mL Pam3CSK4 (Pam3) for 12 h and 20 h, respectively; (**G**,**H**) TNFα and IL-6 levels in the medium of J774A.1 cells 24 h following 50 ng/mL Pam3 stimulation; (**I**–**K**) mRNA expression and; (**L**–**N**) Protein secretion levels of TNFα, IL-6, and IL-1β in THP-1 cells stimulated with 1 ng/mL Pam2CSK4 (Pam2) for 6 h and 20 h, respectively; (**O**,**P**) TNFα and IL-6 levels in the medium of J774A.1 cells 24 h following 25 ng/mL Pam2 stimulation. mRNA expression was measured by qPCR (n = 4–6), and secreted cytokines in the medium were measured by ELISA (n = 8–10); ns: not significant, * *p* < 0.05, ** *p* < 0.01, *** *p* < 0.001, **** *p* < 0.0001.

The putative anti-inflammatory effect of MBSFL and C12:0 was further investigated by the evaluation of their potential to suppress proinflammatory cytokines expression and secretion induced by either a TLR2-TLR1 ligand, Pam3CSK4 (Pam3), or a TLR2-TLR6 ligand, Pam2CSK4 (Pam2). MBSFL treatment significantly reduced Pam3-induced TNFα cytokine secretion in both THP-1 and J774A.1 cells (Figure 2D,G), as well as Pam2-induced TNFα mRNA expression in THP-1 cells and secreted protein levels in J774A.1 cells (Figure 2I,O). MBSFL treatment additionally resulted in decreased IL-6 and IL-1β secretion induced by Pam3 in THP-1 cells (Figure 2E,F). In contrast, LA and SL did not decrease TNFα protein secretion in Pam3- or Pam2-stimulated THP-1 and J774A.1 cells (Figure 2D,G,L). Moreover, LA increased TNFα levels in Pam2-stimulated J774A.1 cells (Figure 2O). The sole observed decrease was in TNFα mRNA expression in Pam2-stimulated THP-1 cells (attributable to LA and SL), which did not correspond with the overall obtained results (Figure 2I). Additionally, treatment with LA or SL increased IL-6 secretion in Pam3-stimulated THP-1 cells as well as IL-6 mRNA expression and protein secretion in Pam2-stimulated THP-1 cells compared to the control. IL-6-induced mRNA expression and protein secretion were partially significant in both Pam3- and Pam2-stimulated THP-1 cells when compared to MBSFL treatment, suggesting a reciprocal modulation effect of MBSFL vs. C12:0 in TLR2-mediated signaling (Figure 2B,E,J,M). 

### 2.3. Treatment with MBSFL or SL Differentially Modifies the Transcriptome Profile of LPS or Pam3-Stimulated THP-1 Cells

**Figure 3:**
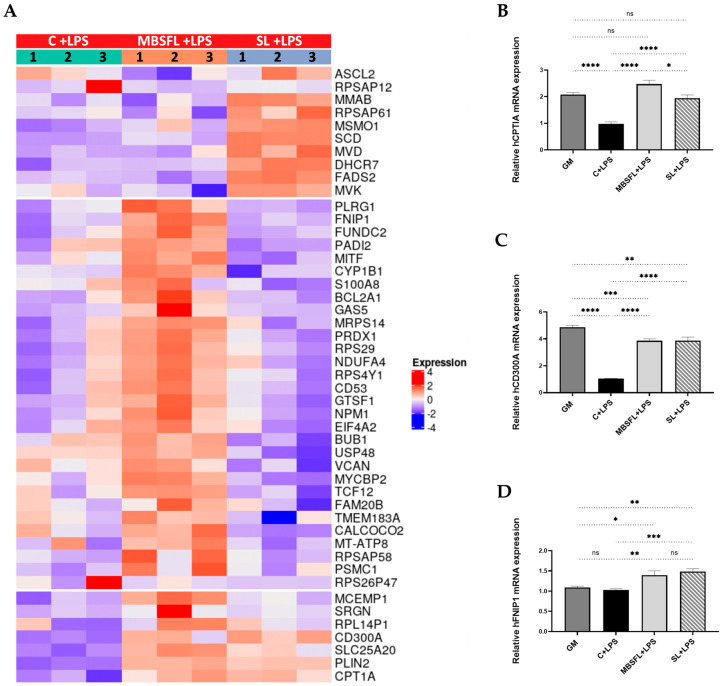

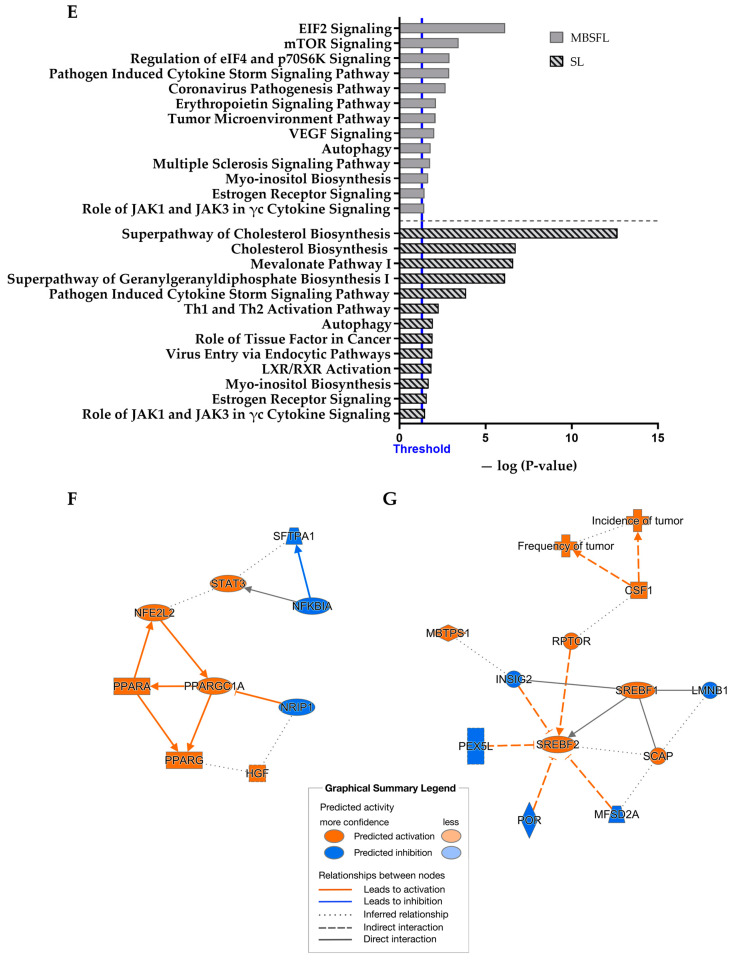
Differential transcriptome profile upon treatment with MBSFL or SL in TLR4-activated macrophages. PMA-primed THP-1 cells were treated with 250 µM MBSFL or 250 µM SL, followed by stimulation with 10 ng/mL LPS for 12 h and compared to stimulated vehicle-treated control (C + LPS). (**A**) Heatmap of Differentially Expressed Genes (DEGs). The log 2-normalized counts were standardized to have a zero mean and standard unit variance for each gene. The standardized log counts were used for clustering analysis. The expression profile is accompanied by a color bar indicating the standardized log 2-normalized counts; (**B**–**D**) mRNA expression of selected DEGs, assessed by qPCR. Data are indicated as mean ± SEM (n = 4–6); ns: not significant, * *p* < 0.05, ** *p* < 0.01, *** *p* < 0.001, **** *p* < 0.0001; (**E**) Lists of selected canonical pathways affected by MBSFL or SL treatment; (**F**,**G**) Graphical interaction networks of the major biological themes and their predicted relationship affected by MBSFL or SL, respectively, based on Ingenuity Pathway Analysis (IPA), using the threshold of fold changes ≥ 1.6 and *p* < 0.01.

To identify the effect of MBSFL on TLR-mediated proinflammatory signaling pathways compared to C12:0, we performed RNA sequencing (RNA-seq) analysis on PMA-primed THP-1 cells stimulated with LPS or Pam3 in the presence or absence of MBSFL or SL. The transcriptomic analysis identified that, in LPS-stimulated cells, treatment with MBSFL or SL essentially resulted in the upregulation of 27 Differentially Expressed Genes (DEGs) and 25 DEGs, respectively. Among them, the peroxisome proliferator-activated receptor (PPAR) β/δ directed target genes *Carnitine palmitoyltransferase 1A* (*CPT1A*) and *CD300A* were upregulated by both MBSFL and SL (Figure 3A). qPCR analysis validated the RNA-seq results and further showed that the expression of these genes was downregulated by LPS; however, MBSFL and SL partially restored their expression to control levels (Figure 3B,C). The gene expression of the metabolic regulator *folliculin interacting protein 1* (*FNIP1*) was increased by MBSFL and SL treatment as well (Figure 3A,D). Amongst the highest-ranking pathways regulated by MBSFL were EIF2, mitotic roles of polo-like kinase (PLK), and EIF4-p70S6K pathways (Figure 3E, Supplementary Table S4A). The graphical summaries show the possible network effect of MBSFL treatment in activating peroxisome proliferator-activated receptor-gamma coactivator (PGC) 1α-PPAR pathway in loop with the activation of the *NFE2-Like BZIP Transcription Factor 2* (*NFE2L2*) gene, which encodes the nuclear factor erythroid 2-related factor 2 (NRF2), as well as the downregulation of *nuclear receptor-interacting protein 1* (*NRIP1*, aka *RIP140*) (Figure 3F). Nonetheless, interestingly, LPS-stimulated macrophages treated with SL predicted the activation of *sterol regulatory element binding transcription Factor 2* (*SREBF2*, encodes SREBP2) at the core of gene interaction, predominantly enriching the cholesterol biosynthesis pathway (Figure 3E,G).

**Figure 4:**
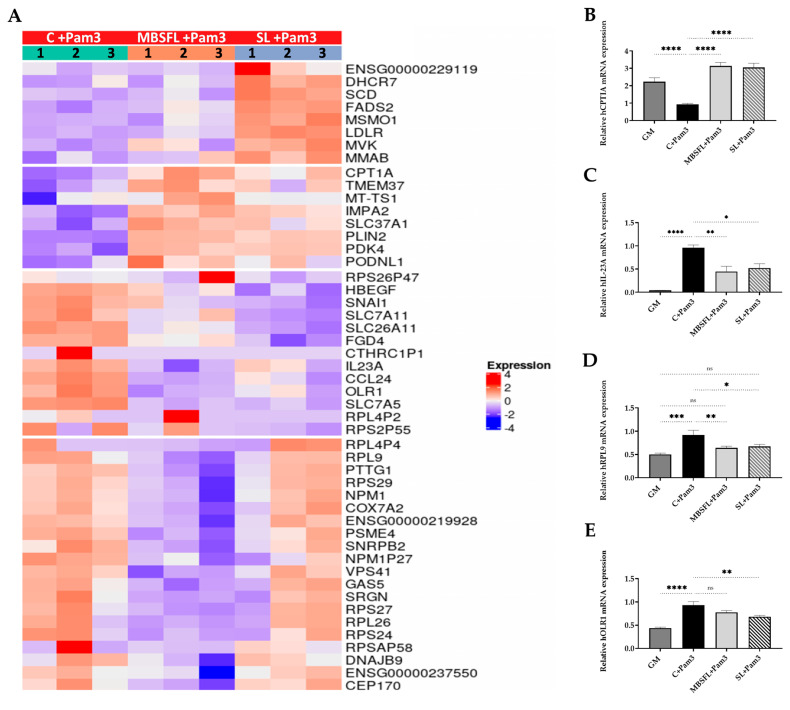

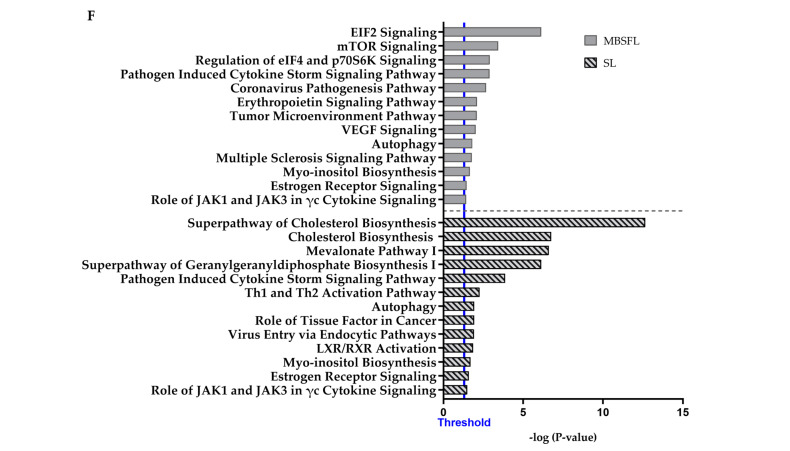

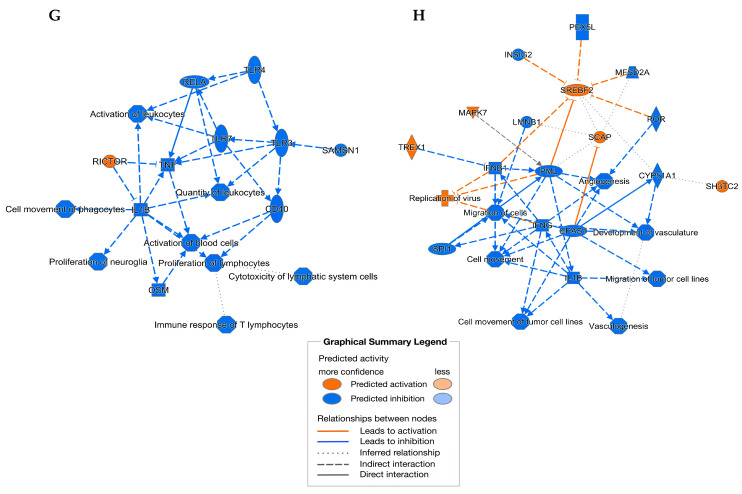
Differential transcriptome profile upon treatment with MBSFL or SL in TLR2-activated macrophages. PMA-primed THP-1 cells were treated with 250 µM MBSFL or 250 µM SL, followed by stimulation with 50 ng/mL Pam3 for 12 h and compared to stimulated vehicle-treated control (C + Pam3). (**A**) Heatmap of DEGs. The log 2-normalized counts were standardized to have a zero mean and standard unit variance for each gene. The standardized log counts were used for the clustering analysis. The expression profile is accompanied by a color bar indicating the standardized log 2-normalized counts; (**B**–**E**) mRNA expression of selected DEGs, assessed by qPCR. Data are indicated as mean ± SEM (n = 4–6); ns: not significant, * *p* < 0.05, ** *p* < 0.01, *** *p* < 0.001, **** *p* < 0.0001; (**F**) Lists of selected canonical pathways affected by MBSFL or SL treatment; (**G**,**H**) Graphical interaction network of the major biological themes and their predicted relationship affected by MBSFL or SL, respectively, based on IPA, using the threshold of fold changes ≥ 1.6 and *p* < 0.01.

Transcriptomic analysis of Pam3-stimulated PMA-primed THP-1 cells identified that MBSFL treatment resulted in 47 DEGs, of which 37 were downregulated and 11 were upregulated. SL treated macrophages exhibited 53 DEGs, of which 27 were downregulated and 26 genes were upregulated, compared to the stimulated but untreated cells in the control group. MBSFL treatment reduced the transcript levels of genes involved in mTOR signaling (encoding ribosomal proteins *RPS27, RPS29*) and genes encoding proinflammatory stimulators (*IL-23*, *nucleophosmin 1* (*NPM1*), *pituitary tumor transforming gene 1* (*PTTG1*), *ribosomal protein 9* (*RPL9*), and *serglycin* (*SRGN*)), as well as genes involved in promoting macrophage polarization toward an M1 phenotype (*Oxidized low-density lipoprotein receptor 1* (*OLR1*) and long noncoding RNA *GAS5*). Among the genes that were upregulated are PPAR direct target genes *CPT1A*, *PLIN2,* and *pyruvate dehydrogenase Kinase 4* (*PDK4*) (Figure 4A). The DEGs of interest, which were involved in different biological processes, were further validated using qPCR (Figure 4B–E). The results of canonical Ingenuity Pathway Analysis (IPA) indicated that MBSFL could be involved in 284 canonical pathways, with the EIF2 signaling and mTOR signaling pathways found to be the highest-ranking signaling pathways (Figure 4F, Supplementary Table S4B). Graphical summaries of the IPA network showed that the anti-inflammatory network effect resulting from MBSFL supplementation was predicted to be related to biological processes involving the inhibition of TLRs, RELA (NF-κB p65 subunit), TNF, and IL-1β cytokines, as well as the upregulation of RICTOR (the main component of mTORC2) (Figure 4G). In contrast, SL treatment activated signaling pathways involved mainly in cholesterol biosynthesis (Figure 4F), and the associated interaction network map predicted the activation of *SREBF2* to be linked to the downregulation of endothelial Per-Arnt-Sim (PAS) domain protein 1 (EPAS1; aka HIF2A) transcription factor and the downregulation of interferon (IFN) responses (Figure 4H). 

### 2.4. Fatty Acid Composition of the BSFL Oil Is Not Directly Correlated to Its Anti-Inflammatory Effect

**Figure 5:**
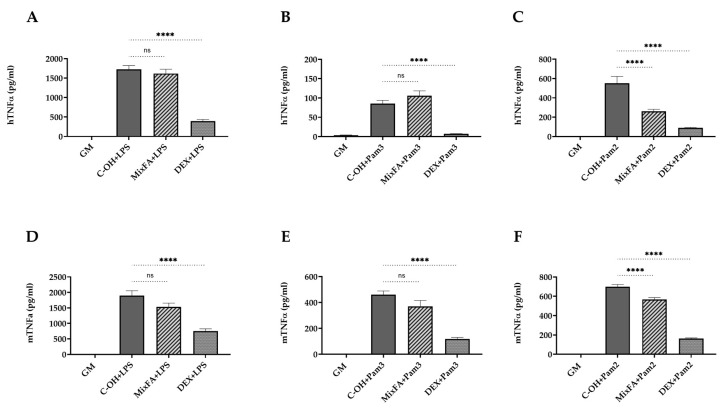
Effect of a synthetic FAs mixture on TNFα secretion in activated macrophages. (**A**–**C**) PMA-primed THP-1 cells were treated with 250 µM FAs mixture (MixFA) or 2 µg/mL DEX, followed by stimulation with 10 ng/mL LPS (**A**), 50 ng/mL Pam3 (**B**), or 1 ng/mL Pam2 (**C**) for 20 h. (**D**–**F**) J774A.1 macrophages were treated with 250 µM MixFA or 1.2 µg/mL DEX, followed by stimulation with 7.5 ng/mL LPS (**D**), 50 ng/mL Pam3 (**E**), or 25 ng/mL Pam2 (**F**) for 24 h. Results were compared to vehicle-treated (C-OH) stimulated controls and untreated and unstimulated cells (GM; Negative control). TNFα in the medium was measured by ELISA (n = 8–10), ns: not significant, **** *p* < 0.0001.

To assess the possible anti-inflammatory effect of BSFL FAs’ unique composition over a single pure C12:0 component, a synthetic FAs mixture that mimics BSFL’s FA profile was prepared. In contrast to MBSFL treatment effect, treatment with the FAs mixture did not reduce TNFα secretion in LPS- or Pam3-stimulated J774A.1 or PMA-primed THP-1 macrophages. Moreover, a significant reduction in TNFα levels by FAs mixture was observed only when J774A.1 and THP-1 cells were stimulated with Pam2 (Figure 5). 

### 2.5. The Ensemble of Anti-Inflammatory Bioactive Lipids Comprising BSFL Oil May Contribute to Its Anti-Inflammatory Activity

The relatively weak correlation between C12:0 content and BSFL oil anti-inflammatory activity led us to search for additional lipid mediators that could potentially contribute to the overall anti-inflammatory characteristics of MBSFL. To this end, we conducted LC–MS analysis to quantify eicosanoid levels since some eicosanoids have been shown to exert pleiotropic signaling functions in metabolic homeostasis and immune responses. We measured the levels of pro-resolving cytochrome P450 (CYP450)-derived ω6 metabolites 9,10-epoxyoctadecenoic acid (9,10-EpOME), 9,10-dihydroxy-12Z-octadecenoic acid (9,10-DiHOME), and 11,12-epoxyeicosatrienoic acid (11,12-EET), the levels of the anti-inflammatory ω3 and ω6 15-lipoxygenase (LOX)-derived 13S-hydroxy-9Z,11E,15Z-octadecatrienoic acid (13S-HOTrE) and 15S-hydroperoxyeicosatetraenoic acid (15S-HpETE), respectively, as well as the anti-inflammatory 12-LOX-derived lipoxin A4 (LXA4) and 12S-hydroxyeicosapentaenoic acid (12S-HEPE) levels. 5-LOX-derived proinflammatory leukotriene (LT) B3 and LTC4 were also detected but only at lower concentrations. Additionally, we identified prostanoids of cyclooxygenase-2 (COX-2)-derived proinflammatory prostaglandin E2 (PGE2) and 6-keto prostaglandin F1α (6-keto PGF1α) on one hand and negative autoregulators PGA1 and PGA2 on the other hand. N-acylamides, oleoyl serotonin (OA-5-HT) and N-acylethanolamines (NAEs) such as stearoyl ethanolamide (SEA), palmitoyl ethanolamide (PEA) and oleoyl ethanolamide (OEA), were also detected (Table 1).

LC–MS analysis for the detection of metabolites associated with the mevalonate pathway in the MBSFL identified the presence of macrophage immune regulatory sterols, squalene, and dolichols. Ubiquinone profile of MBSFL was also determined (Table 2).

### 2.6. Amelioration of Colitis-Associated Indicators in DSS-Treated Mice by BSFL Oil

**Figure 6:**
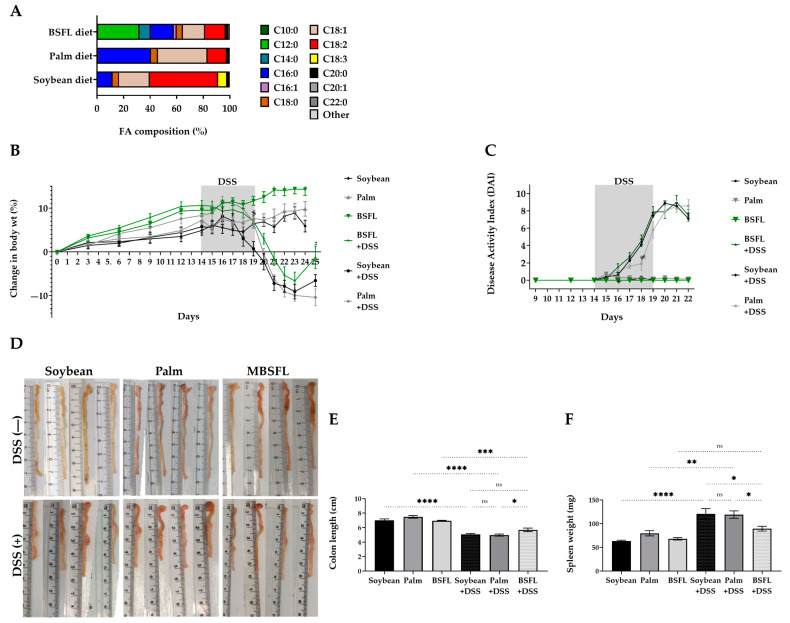
Effect of BSFL oil treatment on the clinical signs of dextran sulfate sodium (DSS)-induced colitis: (**A**) FA composition of diets containing 20% soybean oil, palm oil, or BSFL oil; (**B**) Percentage change in body weight; (**C**) Disease activity index (DAI), a summation of body weight loss, stool consistency, and fecal blood; (**D**) Representative pictures displaying colonic tissues length; (**E**) Changes in colon length; (**F**) Changes in spleen weight. Data presented indicate the mean ± SEM (n = 6–10), ns: not significant, * *p* < 0.05, ** *p* < 0.01, *** *p* < 0.001, **** *p* < 0.0001.

The putative protective role of BSFL oil in the management of inflammatory-related diseases was investigated using a DSS-induced acute colitis mice model, in which the effect of a BSFL oil (20%) diet was evaluated and compared to palm oil or soybean oil diets. The resulting FA compositions of the diets are illustrated in Figure 6A. Within the experimental period (25 days), changes in body weight followed a similar pattern among the various groups (Figure 6B). All mice groups treated with 2.5% DSS showed body weight loss starting from day 3 post DSS administration (day 17), followed by a recovery stage beginning from day 23. However, the extent of weight loss was significantly smaller, and concomitantly the increase in body weight during the recovery period was significantly larger in DSS-treated mice fed with a BSFL oil diet compared with DSS-treated mice fed either with soybean oil or palm oil diets, even though all groups received iso-caloric diets (Supplementary Table S2) and food intake was overall similar among the DSS groups (Supplementary Table S3). Disease activity index (DAI) was measured every day upon DSS administration to quantify the severity of colitis development (Figure 6C). DAI of DSS-treated mice was significantly elevated on day 16 compared to baseline and reached its maximum on day 21. There were no significant differences in DAI among the different dietary DSS groups, except on day 18 of the trial, in which mice fed palm oil diet had a lower DAI score (*p* < 0.05).

Remarkably, the colon length of DSS-treated mice fed with a BSFL oil-based diet was significantly longer compared to DSS-treated mice fed with soybean oil- or palm oil-based diets (*p* < 0.05; Figure 6D,E). Colon shortening as a result of DSS treatment reached 18.8% (*p* < 0.0001) in BSFL oil diet-fed mice in comparison with 27.9% (*p* < 0.0001) and 32.2% (*p* < 0.0001) colon shortening in soybean oil diet-fed mice and palm oil diet-fed mice, respectively, when compared with that of DSS-untreated mice. Furthermore, as shown in Figure 6F, the diet containing BSFL oil showed a protective effect on spleen overactivity, as the spleen weight of mice fed with a BSFL oil-based diet was significantly smaller than the spleen weight of mice fed with soybean oil or palm oil diets (*p* < 0.05). In addition, in comparison with DSS-untreated mice, the spleen weights of DSS-treated mice fed with soybean oil and palm oil diets were significantly larger (90.24%, *p* < 0.0001; 46.54%, *p* < 0.01, respectively) in contrast to DSS-treated mice fed with a BSFL oil-based diet (31.3% increase, a negligible difference).

### 2.7. Histopathological Signs of Colitis in DSS-Treated Mice as a Function of Diet Composition

**Figure 7:**
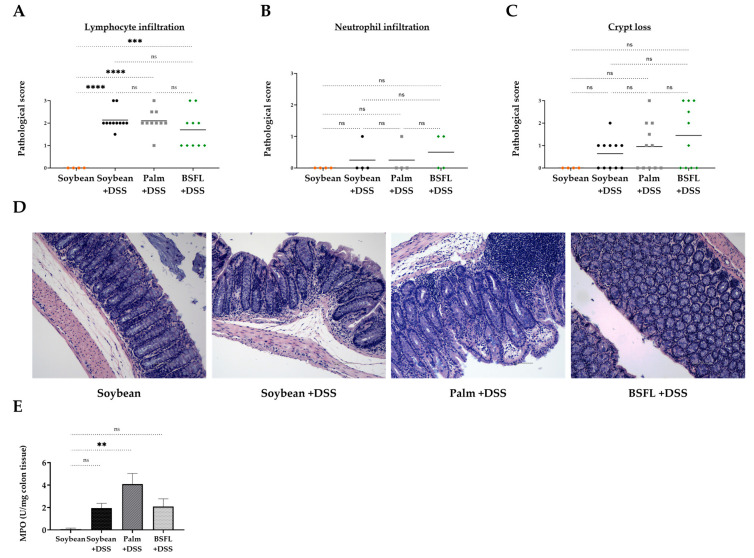
Histologic markers of inflammation and myeloperoxidase (MPO) activity in colon tissues of DSS-induced colitis mice fed with a soybean oil-, palm oil-, or BSFL oil-based diet compared to healthy mice fed with a soybean oil-based diet (control). Colonic histopathological score of (**A**) Lymphocyte infiltration; (**B**) Neutrophil infiltration; (**C**) Crypt loss; (**D**) Representative images of hematoxylin and eosin (H&E) staining of colon tissue from each group (original magnification ×10); (**E**) MPO activity in colon tissue. Values are the mean ± SEM (n = 4–10), ns: not significant, ** *p* < 0.01, *** *p* < 0.001, **** *p* < 0.0001.

Histological analysis revealed an increase in the infiltration of lymphocyte cells in the colonic mucosa, which was induced by DSS treatment and unaffected by the type of diet consumed (Figure 7A). There were no significant differences in neutrophil infiltration (Figure 7B) and crypt loss (Figure 7C,D), projecting the possible consequences of the 5-day recovery period used in the present study. Myeloperoxidase (MPO) activity, a marker of neutrophil influx into the tissue, although repeated a similar pattern of insignificance between the different DSS-treated groups, increased significantly in the DSS-treated mice fed with palm oil when compared to that of the control group (Figure 7E). 

### 2.8. BSFL Oil Induces an Enhanced Earlier Expression of Secretory IgA in Response to DSS Treatment

**Figure 8:**
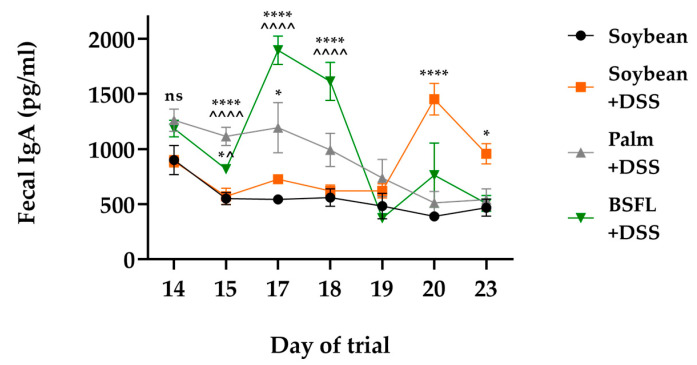
Time-dependent fecal immunoglobulin A (IgA) concentrations of mice fed with BSFL oil, soybean oil, or palm oil diet and treated with DSS compared to healthy mice fed according to a soybean oil-based diet (control). Values are the mean ± SEM (n = 7–10); ns: not significant, * *p* < 0.05, **** *p* < 0.0001 relative to control, ^ *p* < 0.05, ^^^^ *p* < 0.0001 relative to soybean oil+ DSS.

To examine whether dietarily administered BSFL oil could affect intestinal IgA production in DSS-induced colitis, we measured the amounts of fecal IgA released as a function of time. Figure 8 illustrates that fecal IgA release increased in all DSS-treated mice groups compared to IgA values detected in healthy mice fed a control diet. However, mice fed according to a BSFL oil-based diet showed an earlier response of intestinal IgA production, with significantly increased levels of fecal IgA as early as just 24–72 h following DSS administration, compared to DSS-treated mice fed according to soybean oil- or palm oil-based diets. 

## 3. Discussion

This study aimed to evaluate the potential of BSFL oil extract in functioning as a novel putative immunomodulator for the amelioration of IBD based on the notion that this oil extract is rich in lauric acid, which has been reported to exert antimicrobial effects on gut bacteria [12] and anti-inflammatory activity in experimental models in vivo [16,17]. To our knowledge, the role of BSFL oil in inflammation has never been studied until now.

Macrophages are resident cells of almost every tissue in the body and provide key orchestrators of chronic inflammatory disorders. Macrophages have been reported to play a role in the pathological progression of UC disease in comparison with other leukocytes [23]; thus, this evidence prompted us to explore the potential anti-inflammatory effect of MBSFL in murine J774A.1 and PMA-primed human THP-1 macrophages stimulated with various TLR ligands, as a model of inflammation in vitro. Overall, the results indicate that MBSFL exerts a distinct modulatory effect in activated macrophages, possibly dependent on both TLR4 and TLR2 signaling. This premise is based on the finding that MBSFL treatment resulted in a clear reduction in TNFα, IL-6, and IL-1β secretion in LPS-stimulated macrophages, as well as a reduction in secreted TNFα and IL-6 in Pam3-stimulated macrophages. In comparison, C12:0 LA and SL suppressed the release of proinflammatory cytokines TNFα, IL-6, and IL-1β only in LPS-stimulated PMA-primed THP-1 and J774A.1 macrophages. These results are in agreement with those described by Nishimura et al. [24], who showed that LA suppressed TNFα, IL-1β, and IL-6 cytokine production by LPS-stimulated primary microglia. It is worth noting that C12:0 has been reported to promote both TLR4- and TLR2-mediated inflammation, but only in resting, non-activated macrophage cell lines [25,26,27]. BSFL oil contains 42% C12:0, and although both LA and SL suppressed the release of proinflammatory cytokines in TLR4-activated macrophages, LA and SL treatment of TLR2-activated PMA-primed THP-1 cells failed to decrease TNFα secreted levels and resulted in increased IL-6 mRNA expression and protein secretion compared to both control and MBSFL treated cells. Therefore, these discrepant findings were further investigated and clarified using RNA-seq. 

Transcriptome analysis of TLR-activated macrophages in the presence or absence of MBSFL supported its anti-inflammatory effect. In Pam3-stimulated PMA-primed THP-1 cells treated with MBSFL, a significant downregulation of the expression of genes involved in macrophage pro-inflammatory activation was predicted, along with a shift in lipid-glucose metabolism. MBSFL treatment reduced the transcription levels of various proinflammatory stimulators: the proinflammatory cytokine *IL-23A*, *NPM1*, which, when secreted by macrophages, binds TLR4 and promotes TNFα production [28] and the inflammation-related oncogene *PTTG1* [29] and *SRGN* and *RPL9*, whose knockouts were reported to decrease NF-κB activation in vitro [30,31]. The genes encoding ribosomal proteins *RPS27* and *RPS29* were also reduced by MBSFL treatment. The inhibition of mTORC1 is thought to result in the downregulation of genes encoding ribosomal proteins in the 60S and 40S subunits, including *RPS27* [32]. Furthermore, MBSFL treatment resulted in a decreased expression of M1-associated macrophage markers such as *OLR1* [33] and *GAS5* [34]. The anti-inflammatory network effect resulting from MBSFL supplementation was predicted to be related to biological processes involving the inhibition of TLRs and RELA transcription factor and upregulation of RICTOR, suggesting alterations in EIF2 signaling and mTOR signaling. The downregulation of the PI3K/AKT/mTOR pathway was reported to suppress M1 macrophage polarization, thus ameliorating DSS-induced UC in the mice [35]. Concurrently, MBSFL treatment resulted in an increased expression of PPAR direct target genes *PDK4* and *CPT1A*, the latter of which is the rate-limiting enzyme in FAO. The suggested increase in FAO coincides with the observed decrease in the expression of genes associated with M1 activation, as M2-like macrophages are reported to utilize FAO while resolving inflammation induced by PPARγ and controlled by mTORC2 [10]. These results can provide a new and interesting research direction for future studies, potentially leading to investigations into the role of MBSFL in immunometabolism involving macrophage polarization switch and the control of mTOR signaling. In LPS-stimulated macrophages, a similar pattern involving the upregulation of PPAR direct target genes, such as *CPT1A*, *SLC25A20,* and *CD300A*, by MBSFL treatment was observed. PPARβ/δ activation of *CD300A* was previously reported to inhibit TLR4 signaling [36]. A PPARα/δ agonist, lanifibranor, was reported to reduce the expression of proinflammatory mediators while upregulating genes involved in lipid metabolism in hepatic metabolically activated macrophages (derived from infiltrating monocytes) [37]. Taken into consideration that PPARs are nuclear receptors that bind FAs and integrate metabolic and inflammatory signaling pathways [38], a pathway predicted by our results of the gene interaction network map to be affected by MBSFL treatment, it is important to further validate the potential role of MBSFL as a PPAR agonist in future studies. An analysis of the IPA network showed that the possible role of MBSFL in the activation of PPARα and PPARƴ is seemingly linked to the activation of PGC1α and the NRF2 coding gene *NFE2L2*, as well as the downregulation of the RIP140 (NRIP1) transcription regulator. PGC1α was reported to counteract the induction of inflammation by reducing the activity of NF-κB [39] and to promote mitochondrial antioxidant enzyme expression through the *NFE2L2* pathway [40]. RIP140 antagonizes PGC1α and PGC1β activity [41], and its overexpression was reported to inhibit mitochondrial ATP production, enhance glycogenesis, promote the translocation of NF-κB, and increase the production of proinflammatory cytokines in vitro [42]. Therefore, the predicted alterations in the expression of these genes induced by MBSFL treatment further support the anti-inflammatory effect of MBSFL in LPS-stimulated macrophages.

The possible role of MBSFL in immunometabolism switch was not proven to be attributed to its high C12:0 content. DEGs and bioinformatics analysis showed that treating LPS- or Pam3-stimulated PMA-primed THP-1 cells with SL induced the upregulation of pathways mainly involved in cholesterol biosynthesis, presumably mediated via the *SREBF2*-induced upregulation and downregulation of IFN responses, clearly proposing a different mechanism of action in comparison with the treatment effect of MBSFL. Macrophages are reported to shift their cholesterol metabolism in a context-specific manner to ensure the generation of inflammation. Supporting this view, SL shares a TLR2 agonistic character, as TLR2 agonists are reported to activate MyD88-dependent AKT/mTOR signaling, resulting in increased SREBP2 transcriptional activity and cholesterol biosynthesis, while contrasting type I and II IFN signaling [43]. This predicted signaling may partially explain our results of increased proinflammatory cytokines secretion in TLR2-activated THP-1 macrophages treated with SL. Whether C12:0 is a weak agonist of TLR2 remains an intriguing question. Furthermore, these findings challenge a paradigm by which C12:0 contributes to MBSFL potency to modulate TLR-mediated proinflammatory signaling. 

LC–MS analysis for the detection of potent bioactive lipid metabolites resulted in a broad array of potent bioactive eicosanoids, oxylipins, and metabolites of the mevalonate pathway, providing a new perspective in BSFL oil analyses. These metabolites include the CYP450-derived LA and AA epoxy-oxylipins, which have already been proven to play a role in monocyte lineage recruitment and resolution activity during inflammatory resolution [44]. Specifically, 9,10-DiHOME and 9,10-EpOME have been studied for their dual association in inflammation and negative feedback that limits inflammation and pain perception [45]. The following were also detected: 15-LOX-derived ω3 13S-HOTrE which was reported to reduce IL-1β secretion and increase IL-10 secretion [46], 15-LOX-derived ω6 15S-HpETE which was reported to down regulate TNFα, and 12-LOX-derived LXA4 and 12SHEPE which were reported to exert anti-inflammatory effects. In addition, the presence of N-acylserotonin OA-5-HT and NAEs such as SEA, PEA, and OEA, which are key fundamental signaling molecules that have been shown to possess anti-inflammatory properties in vivo [47,48,49], may also potentially contribute to MBSFL anti-inflammatory activity. N-linoleoyl leucine, N-oleoyl valine, and N-palmitoyl glycine were also detected; however, their role in inflammation is yet to be elucidated. Contributing to the complexity of the ensembled eicosanoids detected in MBSFL, we identified molecules opposing regulatory actioned PGs, such as PGE2 and 6-keto PGF1α, molecules demonstrated to be involved in processes leading to the classic signs of inflammation [50], and additionally PGB2, which has been reported to modulate NO production in LPS-stimulated RAW264.7 [51], and PGA1, which has been reported to directly inhibit IKK (leaving NF-κB inactive) [52]. We also identified metabolites of the mevalonate pathway that could serve as potential anti-inflammatory mediators within the MBSFL: Ubiquinones, squalene which was reported to modulate over-activation of monocytes and macrophages [53], and sterol intermediates such as lanosterol, an endogenous immune regulator of macrophages in response to inflammatory stimuli [54], and desmosterol, dihydrolanosterol, and zymosterol which have been proven to activate lipid X receptors (LXRs) [55], i.e., negative regulators of macrophage inflammatory gene expression [56]. To our knowledge, this study is the first of its kind to identify the presence of potent bioactive metabolites produced by BSF during its larval stage; therefore, comparative data from the literature are absent, and the actual contribution of these specific intermediate compounds to the anti-inflammatory activity of BSFL oil warrants further study.

In our in vivo experiment, we showed that BSFL oil improved the clinical manifestations of experimental DSS-induced acute colitis. The efficacy of administration of a diet rich in BSFL oil (20%) for 25 days was demonstrated in C57BL/6 mice treated with 2.5% DSS during days 14–19 of the trial. A BSFL oil-based diet improved the body weight gain during the recovery phase of induced colitis and ameliorated some of the predominant clinical symptoms of colitis, such as reduced colon length and enlarged spleen. Interestingly, among the DSS-treated groups, a different pattern of time-dependent increased fecal IgA secretion was measured as follows: Mice fed according to a BSFL oil-based diet showed an early response of intestinal IgA production that was significantly higher over the period of DSS administration compared to DSS-treated mice fed according to soybean oil- or palm oil-based diet. Increased fecal IgA content is related to improved gut mucosal immune response [57], and Moon et al. demonstrated that mice with decreased levels of IgA were more susceptible to DSS-induced colitis than those with higher IgA levels [58]. To our knowledge, this study is the first of its kind to use BSFL oil in an in vivo colitis model; therefore, future studies using additional established in vivo models of intestinal inflammation should aim to validate the protective role of BSFL oil in IBD. 

## 4. Materials and Methods

### 4.1. Modified BSFL (MBSFL) Oil

BSFL oil extract containing triglycerides (Entoprotech Ltd., Caesarea, Israel) was saponified, i.e., reacted with potassium hydroxide to produce glycerol and lauric acid salts, as described in a published international patent application WO2020234884A1 [59]. 

### 4.2. Fatty Acid–Albumin Complex

MBSFL, natural lauric acid (Sigma-Aldrich, St. Louis, MO, USA, catalog no. W261416), synthetic sodium dodecanoate (sodium laurate; Sigma-Aldrich, catalog no. L9755), or FA mixture (composition is detailed in Supplementary Table S6) were solubilized in albumin, bovine serum (BSA; Sigma-Aldrich, endotoxin-free fraction V, FA-poor, catalog no. 126579, or heat shock fraction, protease-free, FA-free, and essentially globulin-free, catalog no. A7030) to a concentration of 100 mM at 45 °C for 30 min (stock solution). Dexamethasone (Sigma-Aldrich, catalog no. D4902) was also mixed with BSA to compare treatment conditions. Fresh stock solutions were prepared 24 h before each experiment.

### 4.3. Cell Lines

Murine macrophage J774A.1 cell line (ATCC^®^ TIB67™, Manassas, VA, USA) were maintained in a growth medium composed of Dulbecco’s Modified Eagle’s Medium (DMEM; BI, Kibbutz Beit Haemek, Israel), supplemented with 10% (*v*/*v*) heat inactivated fetal bovine serum (HI-FBS; Gibco, Paisley, UK), 1% (*v*/*v*) penicillin-streptomycin (Sigma-Aldrich), and 0.5% (*v*/*v*) sodium pyruvate (Sigma-Aldrich). Human myeloid leukemia cell line THP-1 (a kind gift from Prof. Gabriel Nussbaum, The Innate Immunity Laboratory, The Hebrew University-Hadassah School of Dental Medicine, Israel) were cultured in RPMI 1640 medium (ATCC^®^ 30-2001™) containing 10% (*v*/*v*) HI-FBS, 1% (*v*/*v*) penicillin-streptomycin and 1% (*v*/*v*) sodium pyruvate. Cells were maintained at 37 °C in a humidified 5% (*v*/*v*) CO_2_ incubator.

### 4.4. Cell Treatment 

THP-1 cells (1 × 10^6^ cells/mL) were seeded onto a 96-well culture plate (Thermo Fisher Scientific Inc., Roskilde, Denmark) for ELISA analysis or a 6-well plate (Eppendorf AG, Hamburg, Germany) for qPCR analysis. In order to induce differentiation, THP-1 cells were primed by 10 ng/mL PMA (Sigma-Aldrich) for 72 h, followed by washing with PMA-free medium prior to treatments. J774A.1 cells (2.85 × 10^5^ cells/mL) were seeded onto a 96-well culture plate (for ELISA analysis) and incubated for 24 h before treatment. Cells were treated with 250 µM MBSFL, 250 µM LA, 250 µM SL, dexamethasone (2 µg/mL in THP-1 or 1.2 µg/mL in J774A.1), or a vehicle treatment (BSA alone (C), or BSA plus ethanol at a final concentration of 0.03% (C-OH)). One hour after treatment, cells were stimulated with 7.5–10 ng/mL LPS from *E. coli* O111:B4 (Sigma-Aldrich, catalog no. L5024), 50 ng/mL a synthetic triacylated lipopeptide, Pam3CSK4 (InvivoGen, San Diego, CA, USA), or a synthetic diacylated lipopeptide, Pam2CSK4 (1 ng/mL in THP-1 or 25 ng/mL in J774A.1; InvivoGen).

### 4.5. Cell Viability Assay

Cell viability was evaluated by using the MTT test. Cells were seeded and treated as described above. At the end of the experiment, supernatants were removed and fresh high glucose DMEM without phenol red (Sartorius, Beit Haemek, Israel) containing 0.25 mg/mL of Thiazolyl Blue tetrazolium bromide (MTT; Thermo Fisher Scientific Inc., catalog no. L11939) was added to each well for 2 h. Finally, insoluble formazan crystals were dissolved in 100 µL dimethyl sulfoxide (DMSO; Bio-Lab Ltd., Jerusalem, Israel) for 15 min, and the absorbance was measured at 570 nm using a microplate reader (Infinite M Plex, Tecan Trading AG, Mannedorf, Switzerland). The viability was determined as the percentage of viable cells in treated cultures compared to the percentage of the control group.

### 4.6. Enzyme-Linked Immunosorbent Assay (ELISA)

To detect secreted TNFα, IL-6, IL-1β, and IL-8 cytokine levels, culture supernatants were collected and analyzed with ELISA kits (Invitrogen, Carlsbad, CA, USA) according to the manufacturer’s instructions.

### 4.7. Reverse Transcription PCR (RT-PCR) and Real-Time PCR (qPCR)

Total RNA was isolated from treated THP-1 cells using Nucleospin RNA II kit (Macherey-Nagel, Duren, Germany) and 1 µg was reverse transcribed with qScript^®^ cDNA Synthesis Kit (Quantabio, Gaithersburg, MD, USA). qPCR amplification was performed using QuantStudio 1 system (Applied Biosystems Inc., Waltham, MA, USA), with Fast SYBR™ Green Master Mix (Applied Biosystems Inc.). Primer-BLAST on-line tool was used to design specific primers for hTNFα, hIL-6, hIL-1β, hIL-8, hRPL9, and hGAPDH. Previously described qPCR primer sequences were used for hCPTIA [60], hCD300A [61], hFNIP1 [62], hIL-23A [63], and hOLR1 [64]. The primers used are listed in Supplementary Table S5. All results were normalized to expression of GAPDH gene. The relative expression levels were calculated using the 2^−ΔΔCT^ relative quantification method. 

### 4.8. RNA Sequencing Protocol and Computational Pipeline

Library construction and sequencing. Total RNA was extracted as detailed above. RNA-seq analysis was conducted by the Crown Genomics institute of the Nancy and Stephen Grand Israel National Center for Personalized Medicine, Weizmann Institute of Science. A bulk adaptation of the MARS-Seq protocol [65,66] was used to generate RNA-Seq libraries to profile the expression of THP-1-treated cells (Supplementary Method S1). Sequence data analysis. Assembly and annotation were performed as described previously [67,68]. Differential analysis was performed using DESeq2 package (v1.26.0, Michael Love (HSPH, Boston, MA, USA), Simon Anders, Wolfgang Huber (EMBL, Heidelberg, Germany)) [69] with the betaPrior, cooks Cutoff, and independent Filtering parameters set to False. Raw *p* values were adjusted for multiple testing using the procedure of Benjamini and Hochberg. Pipeline was run using snakemake [70]. DEGs were determined by a *p*-adj of <0.01 and absolute fold changes > 1.6 and max raw counts > 10. Bioinformatics analysis. PCA, Hierarchical clustering, and K-Means clustering were performed (Supplementary Method S1). Standardized, log 2-normalized counts were used for clustering analysis. Clustering analysis was performed using Rstudio (v3.6.1., Boston, MA, USA). DEGs, heatmaps, canonical pathways, and graphical networks were analyzed using Ingenuity Pathways Analysis (IPA, Qiagen, Redwood City, CA, USA).

### 4.9. Metabolomics

Quantitative analysis of all metabolites was carried out by an external authorized laboratory, Creative Proteomics (Shirley, NY, USA). For the analysis of eicosanoids and oxylipins, a LC–MS based platform was used, as detailed in the Supplementary Method S2. The LC gradient was adapted from Watrous et al. [71]. To quantity isoprenyl phosphate intermediates, isoprenoids, and sterols, UPLC–MS/MS was used, as detailed in Supplementary Method S3.

### 4.10. Animals and DSS Model

Male 8-week-old C57BL/6 mice (Envigo, Rehovot, Israel) weighing 18–22 g were housed in an environmentally controlled room at 22 ± 2.0 °C and 50 ± 5% humidity with a 12 h:12 h light/dark cycle and given food and water ad libitum. After acclimatization for 4 days, 51 mice were randomly divided into 3 diet groups (n = 17): 20% BSFL oil (BioBee Sde Eliyahu Ltd., Kibbutz Sde Eliyahu, Israel)-based diet, 20% palm oil-based diet (Envigo, Madison, WI, USA), or 20% soybean oil-based diet (control group; Envigo). Diet compositions are detailed in Supplementary Table S2. The day of special diet composition administration was defined as day 0. To induce colitis, after 14 days of special diet administration, 10 mice from each diet group were treated with 2.5% *w*/*v* DSS (Alfa Aesar, Tewksbury, MA, USA) via introduction into drinking water for 5 additional days, followed by a 5-day recovery. The remaining 7 mice from each diet group received normal drinking water during the entire trial period and served as control. Control groups and DSS-treated mice were sacrificed on days 24 and 25, respectively. A schematic diagram of the experimental design is illustrated in Supplementary Figure S1. During the first two weeks of the trial, body weight was recorded every 3 days. Following DSS administration, body weight, fecal blood (Beckman Coulter, Fullerton, CA, USA), and stool consistency were determined daily. At the end of the trial, colon length and spleen weight were measured. One part of colon tissue was divided and fixed in 4% formaldehyde (Bio-Lab Ltd.) for pathological examination, and the remaining parts were stored at −80 °C for further analysis. All animal care and experimental procedures were approved by the joint ethics committee of the Hebrew University and Hadassah Medical Center, Israel (approval number: AG-20-16366-4).

### 4.11. Fatty Acid Composition

The prepared diets were analyzed with respect to FA content by an external authorized laboratory Merieux NutriSciences (Milouda & Migal Laboratories, Kiryat Shmona, Israel). The analysis was based on the official methods outlined by Association of Official Analytical Chemists (AOAC)—Official Method 996.06 “Fat (Total, Saturated, and Unsaturated) in Foods” (hydrolytic extraction gas chromatographic method).

### 4.12. Disease Activity Index (DAI)

A summation of body weight loss, stool consistency, and stool blood was calculated as described previously [72]. 

### 4.13. Histological Analysis

Three 0.5 mm sections of the colon were fixed in 4% formaldehyde (Bio-Lab Ltd.), embedded in paraffin, and sectioned at 4 μm thickness. The sections were stained with hematoxylin and eosin (H&E) and examined at 100× magnification via light microscopy. H&E-stained sections from mice were scored (0–3) by a certified pathologist.

### 4.14. Myeloperoxidase (MPO) Assay

The level of colonic MPO was determined according to a published method [73] with slight modifications. The colonic tissue specimen (20–40 mg) was homogenized on ice in potassium phosphate buffer pH 6.5 (Sigma-Aldrich) with 0.5% (*w*/*v*) hexadecyltrimethylammonium bromide (HTAB; Sigma-Aldrich) by three 30 s pulses in a Bead Ruptor (OMNI International, Kennesaw, GA, USA). The homogenate was subjected to three 20 s sonications, each followed by 15 min freeze–thaw cycles. The sample was then centrifuged at 15,000× *g* for 30 min at 4 °C. A total of 7 µL of the supernatant fraction was added in triplicate into a 96-well plate, followed by the addition of 200 μL of o-dianisidine mixture containing 0.0005% (*v*/*v*) H_2_O_2_ (Sigma-Aldrich) to each of the wells, and then absorbance at 450 nm was measured with the above plate reader. MPO activity was measured in units (U) of MPO/mg tissue, where one unit of MPO was defined as the amount needed to degrade 1 μmoL of H_2_O_2_ per minute at room temperature.

### 4.15. Fecal IgA

Fecal samples were extracted according to a method published previously by Yingzi Cong et al. [74]. Fecal IgA was measured using mouse IgA ELISA kits (RayBiotech Life, Inc., Peachtree Corners, GA, USA), according to the manufacturer’s instructions.

### 4.16. Statistical Analysis

Data were presented as means ± SEM. The statistical differences between groups were determined by student’s *t*-test or one-way analysis of variance for multiple comparisons in GraphPad Prism 9 software (GraphPad Software Inc., San Diego, CA, USA). Differences were considered as significant at *p* < 0.05.

## 5. Conclusions

Our study provides a convincing indication of the anti-inflammatory activity of black soldier fly larvae (BSFL) oil in vitro and its potential to ameliorate DSS-induced colitis in vivo. Our results offer insight into BSFL oil’s potential in counteracting the initial events of TLR2 and TLR4 activation for macrophage innate immune function, presumably associated with a macrophage-specific immunometabolism switch. This observed phenomenon is apparently not fully attributed to the BSFL oil’s high FA C12:0 content, as a different mode of effect on the inflammatory signaling cascade was demonstrated. Identified anti-inflammatory eicosanoids, oxylipins, and isoprenoids in BSFL oil may potentially contribute to an orchestrated anti-inflammatory response. We hope that advancing our understanding of the effect of BSFL oil on the reprogramming of intracellular metabolism and subsequent macrophage polarization will help to underly a mechanism of action that could be targeted to normalize metabolic homeostasis in inflammatory disease conditions.

## Figures and Tables

**Table 1:** LC–MS quantitative analysis of eicosanoids in MBSFL.

Group	Compound	Concentration (pg/µL)
ω3 metabolite	13S-hydroxy-octadecadienoic acid (13S-HOTrE)	31,505
	17,18-Dihydroxyeicosatetraenoic acid (17,18-DiHETE)	1654
	12S-hydroxyeicosapentaenoic acid (12S-HEPE)	150
ω6 metabolite	9,10-dihydroxyoctadecenoic acid (9,10-DiHOME)	91,118
	9,10-epoxyoctadecenoic acid (9,10-EpOME)	6390
	12-hydroxyoctadecadienoic acid (12-HODE)	12,058
	11,12-epoxyeicosatrienoic acid (11,12-EET)	224
	Lipoxin A4 (LXA4)	1048
	15S-hydroperoxyeicosatetraenoic acid (15S-HpETE)	989
	5S-hydroxyeicosatetraenoic acid (5S-HETE)	159
Prostanoid	Prostaglandin A1 (PGA1)	330
	Prostaglandin B2 (PGB2)	1348
	Prostaglandin E2 (PGE2)	1338
	6-keto Prostaglandin F1α (6-keto PGF1α)	2898
	Thromboxane B1 (TXB1)	5991
Leukotriene	Leukotriene B3 (LTB3)	427
	Leukotriene C4 (LTC4)	222
N-acylamide	N-Linoleoyl Leucine	74,687
	N-Oleoyl Valine	13,233
	N-Palmitoyl Glycine (PalGly)	10,007
N-acylethanol amine (NAE)	Stearoyl Ethanolamide (SEA)	16,739
	Oleoyl Ethanolamide (OEA)	2608
	Palmitoyl Ethanolamide (PEA)	383
N-acyl serotonin	Oleoyl Serotonin (OA-5-HT)	7954

**Table 2:** LC–MS quantitative analysis of mevalonate pathway metabolites in MBSFL.

Group	Compound	Concentration (nmol/g)
Sterols	Lanosterol	20.40
	Desmosterol	2.17
	7-Dehydrocholesterol (7-DHC)	0.56
	Dihydrolanosterol	0.12
	Zymosterol	2.31
	Zymostenol	0.83
Ubiquinones	CoQ-06	2.02
	CoQ-07	2.76
	CoQ-08	1.89
	CoQ-09	24.19
	CoQ-10	0.68
	CoQ-11	0.03
	Squalene	37.82
	Dolichols (13 to 21 isoprene units)	42.71

## Data Availability

Available upon reasonable request.

## Supplementary Materials

The supporting information can be downloaded at: https://www.mdpi.com/article/10.3390/ijms241310634/s1.

## References

[1] Lu Y., Li X., Liu S., Zhang Y., Zhang D. (2018). Toll-like Receptors and Inflammatory Bowel Disease. Front. Immunol..

[2] Płóciennikowska A., Hromada-Judycka A., Borzęcka K., Kwiatkowska K. (2015). Co-Operation of TLR4 and Raft Proteins in LPS-Induced pro-Inflammatory Signaling. Cell. Mol. Life Sci..

[3] Lancaster G.I., Langley K.G., Berglund N.A., Kammoun H.L., Reibe S., Estevez E., Weir J., Mellett N.A., Pernes G., Conway J.R.W. (2018). Evidence That TLR4 Is Not a Receptor for Saturated Fatty Acids but Mediates Lipid-Induced Inflammation by Reprogramming Macrophage Metabolism. Cell Metab..

[4] Wang Y., Qian Y., Fang Q., Zhong P., Li W., Wang L., Fu W., Zhang Y., Xu Z., Li X. (2017). Saturated Palmitic Acid Induces Myocardial Inflammatory Injuries through Direct Binding to TLR4 Accessory Protein MD2. Nat. Commun..

[5] Nicholas D.A., Zhang K., Hung C., Glasgow S., Aruni A.W., Unternaehrer J., Payne K.J., Langridge W.H.R., De Leon M. (2017). Palmitic Acid Is a Toll-like Receptor 4 Ligand That Induces Human Dendritic Cell Secretion of IL-1β. PLoS ONE.

[6] Hattor Y., Kasai K., Akimoto K., Thiemermann C. (1997). Induction of NO Synthesis by Lipoteichoic Acid from *Staphylococcus aureus* in J774 Macrophages: Involvement of a CD14-Dependent Pathway. Biochem. Biophys. Res. Commun..

[7] Ruysschaert J.M., Lonez C. (2015). Role of Lipid Microdomains in TLR-Mediated Signalling. Biochim. Biophys. Acta Biomembr..

[8] Kang J.Y., Nan X., Jin M.S., Youn S.J., Ryu Y.H., Mah S., Han S.H., Lee H., Paik S.G., Lee J.O. (2009). Recognition of Lipopeptide Patterns by Toll-like Receptor 2-Toll-like Receptor 6 Heterodimer. Immunity.

[9] Acosta-Martinez M., Cabail M.Z. (2022). The PI3K/Akt Pathway in Meta-Inflammation. Int. J. Mol. Sci..

[10] Wu M.M., Wang Q.M., Huang B.Y., Mai C.T., Wang C.L., Wang T.T., Zhang X.J. (2021). Dioscin Ameliorates Murine Ulcerative Colitis by Regulating Macrophage Polarization. Pharmacol. Res..

[11] Ganeshan K., Chawla A. (2014). Metabolic Regulation of Immune Responses. Annu. Rev. Immunol..

[12] Gasco L., Finke M., van Huis A. (2018). Can Diets Containing Insects Promote Animal Health?. J. Insects Food Feed.

[13] Papada E., Kaliora A.C., Gioxari A., Papalois A., Forbes A. (2014). Anti-Inflammatory Effect of Elemental Diets with Different Fat Composition in Experimental Colitis. Br. J. Nutr..

[14] Geng S., Zhu W., Xie C., Li X., Wu J., Liang Z., Xie W., Zhu J., Huang C., Zhu M. (2016). Medium-Chain Triglyceride Ameliorates Insulin Resistance and Inflammation in High Fat Diet-Induced Obese Mice. Eur. J. Nutr..

[15] Bertevello P.L., De Nardi L., Torrinhas R.S., Logullo A.F., Waitzberg D.L. (2012). Partial Replacement of ω-6 Fatty Acids with Medium-Chain Triglycerides, but Not Olive Oil, Improves Colon Cytokine Response and Damage in Experimental Colitis. J. Parenter. Enter. Nutr..

[16] Khan H.U., Aamir K., Jusuf P.R., Sethi G., Sisinthy S.P., Ghildyal R., Arya A. (2021). Lauric Acid Ameliorates Lipopolysaccharide (LPS)-Induced Liver Inflammation by Mediating TLR4/MyD88 Pathway in Sprague Dawley (SD) Rats. Life Sci..

[17] Huang W.C., Tsai T.H., Chuang L.T., Li Y.Y., Zouboulis C.C., Tsai P.J. (2014). Anti-Bacterial and Anti-Inflammatory Properties of Capric Acid against Propionibacterium Acnes: A Comparative Study with Lauric Acid. J. Dermatol. Sci..

[18] Kono H., Fujii H., Asakawa M., Maki A., Amemiya H., Hirai Y., Matsuda M., Yamamoto M. (2004). Medium-Chain Triglycerides Enhance Secretory IgA Expression in Rat Intestine after Administration of Endotoxin. Am. J. Physiol. Gastrointest. Liver Physiol..

[19] Okai S., Usui F., Yokota S., Hori-i Y., Hasegawa M., Nakamura T., Kurosawa M., Okada S., Yamamoto K., Nishiyama E. (2016). High-Affinity Monoclonal IgA Regulates Gut Microbiota and Prevents Colitis in Mice. Nat. Microbiol..

[20] Machate D.J., Figueiredo P.S., Marcelino G., de Cássia Avellaneda Guimarães R., Hiane P.A., Bogo D., Pinheiro V.A.Z., Silva de Oliveira L.C., Pott A. (2020). Fatty Acid Diets: Regulation of Gut Microbiota Composition and Obesity and Its Related Metabolic Dysbiosis. Int. J. Mol. Sci..

[21] Tam J.S.Y. (2021). Toll-like Receptor 4 (TLR4) Antagonists as Potential Therapeutics for Intestinal Inflammation. Indian J. Gastroenterol..

[22] Shmuel-Galia L., Aychek T., Fink A., Porat Z., Zarmi B., Bernshtein B., Brenner O., Jung S., Shai Y. (2016). Neutralization of Pro-inflammatory Monocytes by Targeting TLR2 Dimerization Ameliorates Colitis. EMBO J..

[23] Zhang J., Zhao Y., Hou T., Zeng H., Kalambhe D., Wang B., Shen X., Huang Y. (2020). Macrophage-Based Nanotherapeutic Strategies in Ulcerative Colitis. J. Control. Release.

[24] Nishimura Y., Moriyama M., Kawabe K., Satoh H., Takano K., Azuma Y.T., Nakamura Y. (2018). Lauric Acid Alleviates Neuroinflammatory Responses by Activated Microglia: Involvement of the GPR40-Dependent Pathway. Neurochem. Res..

[25] Lee J.Y., Zhao L., Youn H.S., Weatherill A.R., Tapping R., Feng L., Lee W.H., Fitzgerald K.A., Hwang D.H. (2004). Saturated Fatty Acid Activates but Polyunsaturated Fatty Acid Inhibits Toll-like Receptor 2 Dimerized with Toll-like Receptor 6 or 1. J. Biol. Chem..

[26] Lee J.Y., Ye J., Gao Z., Youn H.S., Lee W.H., Zhao L., Sizemore N., Hwang D.H. (2003). Reciprocal Modulation of Toll-like Receptor-4 Signaling Pathways Involving MyD88 and Phosphatidylinositol 3-Kinase/AKT by Saturated and Polyunsaturated Fatty Acids. J. Biol. Chem..

[27] Huang S., Rutkowsky J.M., Snodgrass R.G., Ono-Moore K.D., Schneider D.A., Newman J.W., Adams S.H., Hwang D.H. (2012). Saturated Fatty Acids Activate TLR-Mediated Proinflammatory Signaling Pathways. J. Lipid Res..

[28] Nakatomi K., Ueno H., Ishikawa Y., Salim R.C., Mori Y., Kanemoto I., Tancharoen S., Kikuchi K., Miura N., Omori T. (2020). Tlr4/Md-2 Is a Receptor for Extracellular Nucleophosmin 1. Biomed. Rep..

[29] Lin X., Yang Y., Guo Y., Liu H., Jiang J., Zheng F., Wu B. (2019). PTTG1 Is Involved in TNF-α-Related Hepatocellular Carcinoma via the Induction of c-Myc. Cancer Med..

[30] Scuruchi M., D’Ascola A., Avenoso A., Mandraffino G.G., Campo S.S., Campo G.M. (2019). Serglycin as Part of IL-1β Induced Inflammation in Human Chondrocytes. Arch. Biochem. Biophys..

[31] El Khoury W., Nasr Z. (2021). Deregulation of Ribosomal Proteins in Human Cancers. Biosci. Rep..

[32] Rosario F.J., Powell T.L., Gupta M.B., Cox L., Jansson T. (2020). MTORC1 Transcriptional Regulation of Ribosome Subunits, Protein Synthesis, and Molecular Transport in Primary Human Trophoblast Cells. Front. Cell Dev. Biol..

[33] Sun X., Fu X., Xu S., Qiu P., Lv Z., Cui M., Zhang Q., Xu Y. (2021). OLR1 Is a Prognostic Factor and Correlated with Immune Infiltration in Breast Cancer. Int. Immunopharmacol..

[34] Hu J., Zhang L., Liechty C., Zgheib C., Hodges M.M., Liechty K.W., Xu J. (2020). Long Noncoding RNA GAS5 Regulates Macrophage Polarization and Diabetic Wound Healing. J. Investig. Dermatol..

[35] Bai D., Zhao Y., Zhu Q., Zhou Y., Zhao Y., Zhang T., Guo Q., Lu N. (2018). LZ205, a Newly Synthesized Flavonoid Compound, Exerts Anti-Inflammatory Effect by Inhibiting M1 Macrophage Polarization through Regulating PI3K/AKT/MTOR Signaling Pathway. Exp. Cell Res..

[36] Tanaka T., Tahara-Hanaoka S., Nabekura T., Ikeda K., Jiang S., Tsutsumi S., Inagaki T., Magoori K., Higurashi T., Takahashi H. (2014). PPARβ 2/Δactivation of CD300a Controls Intestinal Immunity. Sci. Rep..

[37] Lefere S., Puengel T., Hundertmark J., Penners C., Frank A.K., Guillot A., de Muynck K., Heymann F., Adarbes V., Defrêne E. (2020). Differential Effects of Selective- and Pan-PPAR Agonists on Experimental Steatohepatitis and Hepatic Macrophages. J. Hepatol..

[38] Varga T., Czimmerer Z., Nagy L. (2011). PPARs Are a Unique Set of Fatty Acid Regulated Transcription Factors Controlling Both Lipid Metabolism and Inflammation. Biochim. Biophys. Acta Mol. Basis Dis..

[39] Eisele P.S., Furrer R., Beer M., Handschin C. (2015). The PGC-1 Coactivators Promote an Anti-Inflammatory Environment in Skeletal Muscle in Vivo. Biochem. Biophys. Res. Commun..

[40] Cherry A.D., Suliman H.B., Bartz R.R., Piantadosi C.A. (2014). Peroxisome Proliferator-Activated Receptor γ Co-Activator 1-α as a Critical Co-Activator of the Murine Hepatic Oxidative Stress Response and Mitochondrial Biogenesis in *Staphylococcus aureus* Sepsis. J. Biol. Chem..

[41] Nautiyal J., Christian M., Parker M.G. (2013). Distinct Functions for RIP140 in Development, Inflammation, and Metabolism. Trends Endocrinol. Metab..

[42] Guo Z., Shen Y., Zhong J., Li Z., Guo Q., Yao X., Wang Y., Wu W. (2022). RIP140-Mediated NF-ΚB Inflammatory Pathway Promotes Metabolic Dysregulation in Retinal Pigment Epithelium Cells. Curr. Issues Mol. Biol..

[43] Lee M.S., Bensinger S.J. (2022). Reprogramming Cholesterol Metabolism in Macrophages and Its Role in Host Defense against Cholesterol-Dependent Cytolysins. Cell. Mol. Immunol..

[44] Gilroy D.W., Edin M.L., De Maeyer R.P.H., Bystrom J., Newson J., Lih F.B., Stables M., Zeldin D.C., Bishop-Bailey D. (2016). CYP450-Derived Oxylipins Mediate Inflammatory Resolution. Proc. Natl. Acad. Sci. USA.

[45] Hildreth K., Kodani S.D., Hammock B.D., Zhao L. (2020). Cytochrome P450-Derived Linoleic Acid Metabolites EpOMEs and DiHOMEs: A Review of Recent Studies. J. Nutr. Biochem..

[46] Pauls S.D., Rodway L.A., Winter T., Taylor C.G., Zahradka P., Aukema H.M. (2018). Anti-Inflammatory Effects of α-Linolenic Acid in M1-like Macrophages Are Associated with Enhanced Production of Oxylipins from α-Linolenic and Linoleic Acid. J. Nutr. Biochem..

[47] Kasatkina L.A., Heinemann A., Hudz Y.A., Thomas D., Sturm E.M. (2020). Stearoylethanolamide Interferes with Retrograde Endocannabinoid Signalling and Supports the Blood-Brain Barrier Integrity under Acute Systemic Inflammation. Biochem. Pharmacol..

[48] Ezzili C., Otrubova K., Boger D.L. (2010). Fatty Acid Amide Signaling Molecules. Bioorg. Med. Chem. Lett..

[49] Schlosburg J.E., Kinsey S.G., Lichtman A.H. (2009). Targeting Fatty Acid Amide Hydrolase (FAAH) to Treat Pain and Inflammation. AAPS J..

[50] Boughton-Smith N.K., Wallace J.L., Morris G.P., Whittle B.J.R. (1988). The Effect of Anti-inflammatory Drugs on Eicosanoid Formation in a Chronic Model of Inflammatory Bowel Disease in the Rat. Br. J. Pharmacol..

[51] Shen H., Liu X., Jiang M., Luo G., Wu Z., Chen B., Li J., Liu L., Chen S. (2019). Anti-Inflammatory Cembrane-Type Diterpenoids and Prostaglandins from Soft Coral Lobophytum Sarcophytoides. Mar. Drugs.

[52] Fitc F., Tritc R. (2000). Anti-Inflammatory Cyclopentenone Prostaglandins Are Direct Inhibitors of IkB Kinase. Nature.

[53] Cárdeno A., Aparicio-Soto M., Montserrat-de la Paz S., Bermudez B., Muriana F.J.G., Alarcón-de-la-Lastra C. (2015). Squalene Targets Pro- and Anti-Inflammatory Mediators and Pathways to Modulate over-Activation of Neutrophils, Monocytes and Macrophages. J. Funct. Foods.

[54] Araldi E., Fernández-Fuertes M., Canfrán-Duque A., Tang W., Cline G.W., Madrigal-Matute J., Pober J.S., Lasunción M.A., Wu D., Fernández-Hernando C. (2017). Lanosterol Modulates TLR4-Mediated Innate Immune Responses in Macrophages. Cell Rep..

[55] Snodgrass R.G., Benatzy Y., Schmid T., Namgaladze D., Mainka M., Schebb N.H., Lütjohann D., Brüne B. (2021). Efferocytosis Potentiates the Expression of Arachidonate 15-Lipoxygenase (ALOX15) in Alternatively Activated Human Macrophages through LXR Activation. Cell Death Differ..

[56] Joseph S.B., Castrillo A., Laffitte B.A., Mangelsdorf D.J., Tontonoz P. (2003). Reciprocal Regulation of Inflammation and Lipid Metabolism by Liver X Receptors. Nat. Med..

[57] Kang Y., Yang G., Zhang S., Ross C.F., Zhu M.J. (2018). Goji Berry Modulates Gut Microbiota and Alleviates Colitis in IL-10-Deficient Mice. Mol. Nutr. Food Res..

[58] Moon C., Baldridge M.T., Wallace M.A., Burnham C.-A.D., Virgin H.W., Stappenbeck T.S. (2015). Vertically Transmitted Fecal IgA Levels Distinguish Extra-Chromosomal Phenotypic Variation. Nature.

[59] Richter H., Steinberg S., Baron Y., Inbart A. (2020). Modified Black Soldier Fly Larvae Oil with Modified Lauric Acid for Treatment against Biofilm Formation and Microorganism Growth. Internationl Application.

[60] Kohjima M., Enjoji M., Higuchi N., Kato M., Kotoh K., Yoshimoto T., Fujino T., Yada M., Yada R., Harada N. (2007). Re-Evaluation of Fatty Acid Metabolism-Related Gene Expression in Nonalcoholic Fatty Liver Disease. Int. J. Mol. Med..

[61] Ju X., Zenke M., Hart D.N.J., Clark G.J. (2008). CD300a/c Regulate Type i Interferon and TNF-α Secretion by Human Plasmacytoid Dendritic Cells Stimulated with TLR7 and TLR9 Ligands. Blood.

[62] Hasumi H., Baba M., Hong S.B., Hasumi Y., Huang Y., Yao M., Valera V.A., Linehan W.M., Schmidt L.S. (2008). Identification and Characterization of a Novel Folliculin-Interacting Protein FNIP2. Gene.

[63] Hor Y.T., Voon D.C.C., Koo J.K.W., Wang H., Lau W.M., Ashktorab H., Chan S.L., Ito Y. (2014). A Role for RUNX3 in Inflammation-Induced Expression of IL23A in Gastric Epithelial Cells. Cell Rep..

[64] Yamagata K., Tusruta C., Ohtuski A., Tagami M. (2014). Docosahexaenoic Acid Decreases TNF-α-Induced Lectin-like Oxidized Low-Density Lipoprotein Receptor-1 Expression in THP-1 Cells. Prostaglandins Leukot. Essent. Fat. Acids.

[65] Jaitin D.A., Kenigsberg E., Keren-Shaul H., Elefant N., Paul F., Zaretsky I., Mildner A., Cohen N., Jung S., Tanay A. (2014). Massively Parallel Single-Cell RNA-Seq for Marker-Free Decomposition of Tissues into Cell Types. Science.

[66] Keren-Shaul H., Kenigsberg E., Jaitin D.A., David E., Paul F., Tanay A., Amit I. (2019). MARS-Seq2.0: An Experimental and Analytical Pipeline for Indexed Sorting Combined with Single-Cell RNA Sequencing. Nat. Protoc..

[67] Martin M. (2011). Cutadapt Removes Adapter Sequences from High-Throughput Sequencing Reads. EMBnet.J..

[68] Anders S., Pyl P.T., Huber W. (2015). HTSeq-A Python Framework to Work with High-Throughput Sequencing Data. Bioinformatics.

[69] Love M.I., Huber W., Anders S. (2014). Moderated Estimation of Fold Change and Dispersion for RNA-Seq Data with DESeq2. Genome Biol..

[70] Köster J., Rahmann S. (2012). Snakemake—A Scalable Bioinformatics Workflow Engine. Bioinformatics.

[71] Watrous J.D., Niiranen T.J., Lagerborg K.A., Henglin M., Xu Y.J., Rong J., Sharma S., Vasan R.S., Larson M.G., Armando A. (2019). Directed Non-Targeted Mass Spectrometry and Chemical Networking for Discovery of Eicosanoids and Related Oxylipins. Cell Chem. Biol..

[72] Jeengar M.K., Thummuri D., Magnusson M., Naidu V.G.M. (2017). Uridine Ameliorates Dextran Sulfate Sodium (DSS)-Induced Colitis in Mice. Sci. Rep..

[73] Kim J.J., Shajib M.S., Manocha M.M., Khan W.I. (2012). Investigating Intestinal Inflammation in DSS-Induced Model of IBD. J. Vis. Exp..

[74] Cao A.T., Yao S., Gong B., Elson C.O., Cong Y. (2012). Th17 Cells Upregulate Polymeric Ig Receptor and Intestinal IgA and Contribute to Intestinal Homeostasis. J. Immunol..

