# Supplementary material for: Anti-Inflammatory Activity of Black Soldier Fly Oil Associated with Modulation of TLR Signaling: A Metabolomic Approach
Source: Int J Mol Sci. 2023 Jun 25;24(13):10634. doi: 10.3390/ijms241310634 (PMC10341857; doi:10.3390/ijms241310634)
Supplement: Supplementary file 1 [file ijms-24-10634-s001.zip › ijms-2436921-supplementary.pdf]

Article

Anti-Inflammatory Activity of Black Soldier Fly Oil Associated with Modulation of TLR Signaling: A Metabolomic Approach

Hadas Richter, Ofer Gover and Betty Schwartz *

Supplementary information

Supplementary Table S1. Fatty acid profile of BSFL oil.

**Batch No.**	BSFL oil 071220, Entoprotech Ltd.		
**Total saturated acids (% DM)**	67.34		
**Total unsaturated acids (% DM)**	32.53		
**Fatty Acid Profile (%DM)**	C 8:0	Caprylic acid	ND
	C 10:0	Capric acid	1.45
	C 12:0	Lauric acid	42.53
	C 14:0	Myristic acid	7.86
	C 16:0	Palmitic acid	11.91
	C 16:1	Palmitoleic acid	1.76
	C 18:0	Stearic acid	1.84
	C 18:1	Oleic acid	9.72
	C 18:2 n6	Linoleic acid	18.80
	C 18:3 n3	Alpha Linolenic acid	1.800
	C 21:0	Heneicosylic acid	1.177

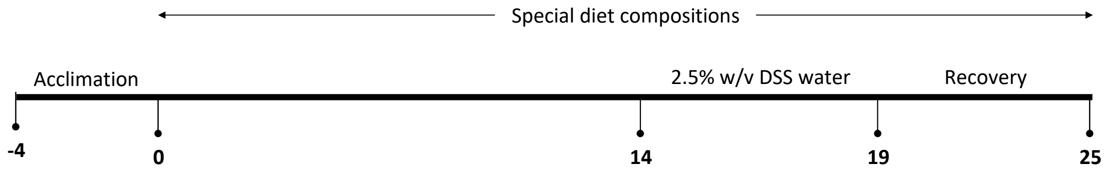

Supplementary Figure S1. Schematic diagram to illustrate the experimental design, the day of special diet composition administration was defined as day 0.

Supplementary Table S2. Chow-based diet compositions used in the study.

Diet Composition		20% Soybean oil	20% palm oil	20% BSFL oil
**Energy (Kcal/100g)**		442	442	428
**Protein (g/100g)**		16.04	15.87	15.96
**Carbohydrate (g/100g)**		47.06	46.74	45.54
**Moisture (g/100g)**		11.81	12.24	14.4
**Ash (g/100g)**		3.87	3.89	3.9
**Fat (g/100g)**		21.12	21.26	20.2
**% Saturated**		3.73	9.920	12.91
**Fatty Acid (g/100g oil)**				
	C 10:0	ND	ND	0.76
	C12:0	0.07	0.198	31.2
	C 14:0	0.11	0.882	8.52
	C16:0	11.41	39.55	17.29
	C16:1 n7	0.10	0.163	2.28
	C18:0	4.96	5.120	4.66
	C18:1	23.15	37.25	16.63
	C 18:2 n6	51.41	14.78	15.63
	C 18:3 n3 ALA	6.84	0.7	0.98
	C 20:0	0.37	0.39	0.54
	C 20:1 n9	0.53	0.24	0.24
	C 22:0	0.374	0.18	0.09

Supplementary Table S3. Average food intake (g/day) of mice fed on different diet compositions throughout the experimental period.

**Food intake** (g/day per mouse)	**Days 0-7** (Week 1)	**Days 8-15** (Week 2)	**Days 16- 24** (Week 3)
Soybean diet	3.2	3.5	3.5
Palm diet	3.0	3.5	3.5
BSFL diet	3.7	3.3	3.5
Soybean diet +DSS	3.6 ± 0.1 ns	4.1 ± 0.2 ns	2.9 ± 0.2 ns
Palm diet +DSS	3.8 ± 0.5 ns	4.1 ± 0.1 ns	2.9 ± 0.1 ns
BSFL diet +DSS	4.2 ± 0.3 ns	4.0 ± 0.04 ns	3.3 ± 0.03 ns

Data are presented as mean ± SEM (n=2), ns: not significant

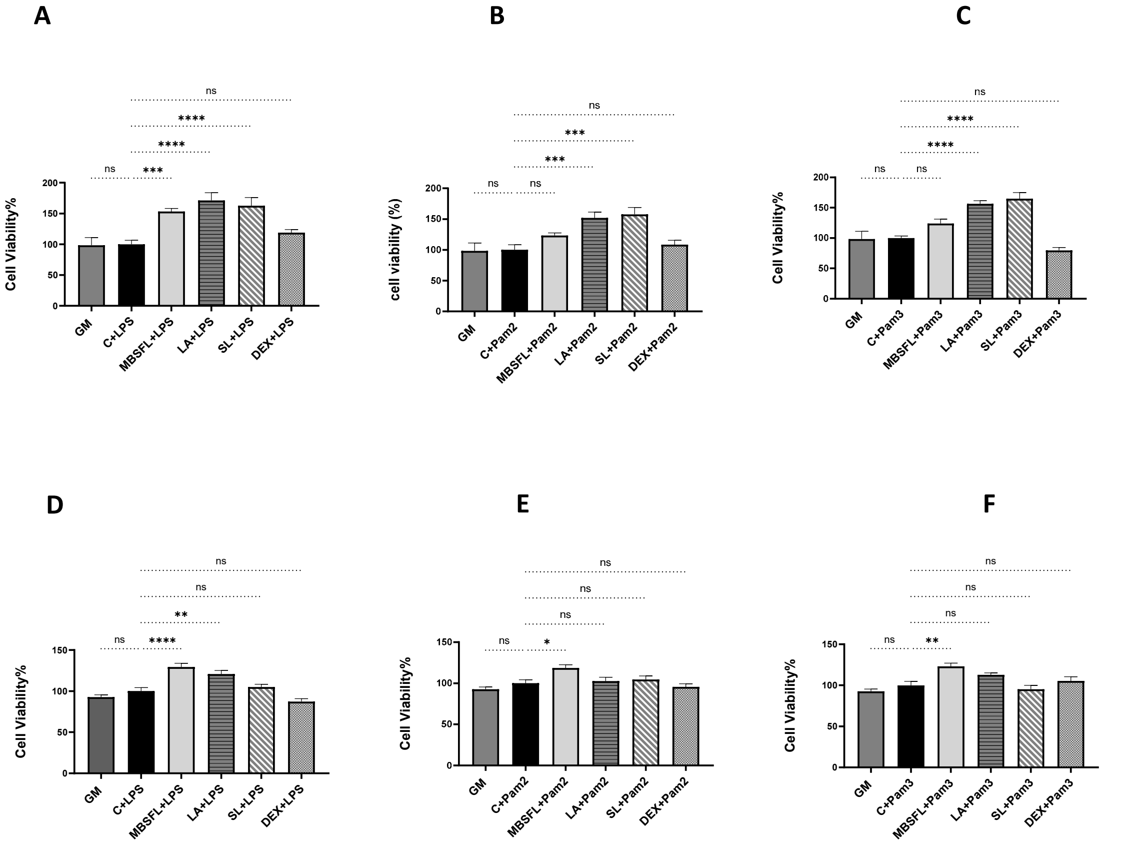

Supplementary Figure S2. Effect of various treatments on cell viability. THP-1 (**A–C**) or J774A.1 (**D–F**) cells were stimulated with 10 ng/mL LPS (Figures S2A,D), 1 or 25 ng/ml Pam2CSK4 (Figures S2B and S2E, respectively), or 50 ng/mL Pam3CSK4 (Figures S2C,F) for 20 or 24 h, respectively, in the presence of 250 μM modified BSFL oil (MBSFL), 250 μM lauric acid (LA) 250 μM sodium laurate (SL) or dexamethasone (DEX; 2 μg/ml in THP-1, 1.2 μg/ml in J774A.1)), and in comparison with vehicle-treated (C) stimulated controls, and untreated and unstimulated cells (GM). Cell viability was assessed with the MTT assay. Data are the mean ± SEM of 8–10 samples. ns: not significant, * $p < 0.05$, ** $p < 0.01$, *** $p < 0.001$, **** $p < 0.0001$ versus stimulated control group (C+LPS, C+Pam2 or C+Pam3).

Supplementary Tables S4A. Canonical pathways affected by MBSFL treatment in LPS- stimulated THP-1.

Ingenuity Canonical Pathways	-log(p-value)	Ratio	Molecules
EIF2 Signaling	5.06	0.0352	EIF1AY,PIK3C3,RPL26,RPL34,RPL9,RPS29,RPS3A,RPS4Y1
Mitotic Roles of Polo-Like Kinase	3.63	0.0597	CDK1,FBXO5,PTTG1,SLK
Docosahexaenoic Acid (DHA) Signaling	3.18	0.0789	BAD,BCL2A1,PIK3C3
Ketolysis	3.06	0.2	ACAA2,OXCT1
Regulation of eIF4 and p70S6K Signaling	2.87	0.0276	EIF1AY,PIK3C3,RPS29,RPS3A,RPS4Y1
FAT10 Signaling Pathway	2.69	0.0536	PSMA1,PSMC1,PSME4
Coronavirus Pathogenesis Pathway	2.64	0.0245	NPM1,PIK3C3,RPS29,RPS3A,RPS4Y1
Hereditary Breast Cancer Signaling	2.41	0.0282	CDK1,NPM1,PIK3C3,SMARCD3
Inhibition of ARE-Mediated mRNA Degradation Pathway	2.21	0.0247	CNOT2,PSMA1,PSMC1,PSME4
BAG2 Signaling Pathway	2.19	0.0357	PSMA1,PSMC1,PSME4
Ribonucleotide Reductase Signaling Pathway	2.14	0.0235	BAD,CDK1,PIK3C3,SMARCD3
Role of p14/p19ARF in Tumor Suppression	2.1	0.0667	NPM1,PIK3C3
VEGF Signaling	2	0.0303	BAD,EIF1AY,PIK3C3
Apoptosis Signaling	1.94	0.0288	BAD,BCL2A1,CDK1
Tetrahydrobiopterin Biosynthesis I	1.87	0.333	GCH1
Tetrahydrobiopterin Biosynthesis II	1.87	0.333	GCH1
mTOR Signaling	1.8	0.0187	PIK3C3,RPS29,RPS3A,RPS4Y1
DNA damage-induced 14-3-3σ Signaling	1.74	0.0435	BAD,CDK1
Melanoma Signaling	1.67	0.04	BAD,PIK3C3
Galactose Degradation I (Leloir Pathway)	1.65	0.2	GALM
UVB-Induced MAPK Signaling	1.64	0.0385	BAD,PIK3C3
CSDE1 Signaling Pathway	1.58	0.0357	CDK11B,RPL9
Cell Cycle Control of Chromosomal Replication	1.58	0.0357	CDK1,CDK11B
Endometrial Cancer Signaling	1.52	0.0333	BAD,PIK3C3
Phagosome Maturation	1.46	0.019	PIK3C3,PRDX1,RAB5B
ERB2-ERBB3 Signaling	1.46	0.0308	BAD,PIK3C3
Huntington's Disease Signaling	1.41	0.0141	PIK3C3,PSMA1,PSMC1,PSME4
GM-CSF Signaling	1.4	0.0286	BCL2A1,PIK3C3
Salvage Pathways of Pyrimidine Deoxyribonucleotides	1.4	0.111	APOBEC3A
Granzyme A Signaling	1.35	0.0267	MT-ND6,NDUFA4
Angiopoietin Signaling	1.34	0.0263	BAD,PIK3C3
Glucocorticoid Receptor Signaling	1.32	0.0103	KRT17,MT-ND6,NDUFA4,PDK4,PIK3C3,SMARCD3
IL-7 Signaling Pathway	1.32	0.0256	BAD,PIK3C3
Ketogenesis	1.32	0.0909	ACAA2
Sucrose Degradation V (Mammalian)	1.32	0.0909	GALM
IL-3 Signaling	1.31	0.0253	BAD,PIK3C3
FLT3 Signaling in Hematopoietic Progenitor Cells	1.28	0.0244	BAD,PIK3C3
Oleate Biosynthesis II (Animals)	1.25	0.0769	UFSP2

Supplementary Table S4B. Canonical pathways affected by MBSFL treatment in Pam3CSK4-stimulated THP-1.

Ingenuity Canonical Pathways	-log(p-value)	Ratio	Molecules
EIF2 Signaling	6.13	0.0396	EIF1AY,RPL26,RPL34,RPL9,RPS24,RPS27,RPS29,RPS4Y1,VEGFA
mTOR Signaling	3.43	0.028	PRKD3,RPS24,RPS27,RPS29,RPS4Y1,VEGFA
Regulation of eIF4 and p70S6K Signaling	2.9	0.0276	EIF1AY,RPS24,RPS27,RPS29,RPS4Y1
Pathogen Induced Cytokine Storm Signaling Pathway	2.89	0.0189	BHLHE40,CCL24,IL12B,IL23A,SLC2A1,SRGN,VEGFA
Coronavirus Pathogenesis Pathway	2.68	0.0245	NPM1,RPS24,RPS27,RPS29,RPS4Y1
Role of Hypercytokinemia/hyperchemokinemia in the Pathogenesis of Influenza	2.19	0.0349	IFIT3,IL12B,ISG20
RANK Signaling in Osteoclasts	2.12	0.033	BIRC3,SRC,TAB2
Erythropoietin Signaling Pathway	2.1	0.0226	BIRC3,IL12B,PRKD3,SRC
Tumor Microenvironment Pathway	2.09	0.0223	SLC1A4,SLC2A1,TNC,VEGFA
Airway Inflammation in Asthma	2.03	0.0606	IL12B,IL23A
VEGF Signaling	2.02	0.0303	EIF1AY,SRC,VEGFA
Sirtuin Signaling Pathway	2.02	0.0171	CPT1A,NDUFA4,NDUFB3,SLC25A4,SLC2A1
Neutrophil Extracellular Trap Signaling Pathway	2	0.0145	NDUFA4,NDUFB3,PRKD3,SLC25A4,SLC2A1,SRC
Oxidative Phosphorylation	1.88	0.027	COX7A2,NDUFA4,NDUFB3
Autophagy	1.81	0.0185	SLC7A5,VEGFA,VPS41,WIPI1
Multiple Sclerosis Signaling Pathway	1.77	0.018	GAS5,IL12B,IL23A,IL7R
IL-23 Signaling Pathway	1.76	0.0435	IL12B,IL23A
IL-12 Signaling and Production in Macrophages	1.69	0.0169	IL12B,IL23A,PRKD3,S100A8
S100 Family Signaling Pathway	1.68	0.0104	ADGRE1,CYSLTR2,IL12B,IL23A,PRKD3,S100A8,SRC,VEGFA
UVC-Induced MAPK Signaling	1.67	0.0392	PRKD3,SRC
Protein Citrullination	1.66	0.2	PADI2
Myo-inositol Biosynthesis	1.66	0.2	IMPA2
STAT3 Pathway	1.66	0.0222	IL7R,SRC,VEGFA
Role of Cytokines in Mediating Communication between Immune Cells	1.63	0.037	IL12B,IL23A
CSDE1 Signaling Pathway	1.6	0.0357	RPL9,TNC
Chondroitin and Dermatan Biosynthesis	1.58	0.167	CHSY1
Role of JAK2 in Hormone-like Cytokine Signaling	1.51	0.0323	BIRC3,VEGFA
Necroptosis Signaling Pathway	1.5	0.0192	BIRC3,SLC25A4,TAB2
Protein Ubiquitination Pathway	1.48	0.0147	BIRC3,DNAJB9,USP37,USP47
Induction of Apoptosis by HIV1	1.48	0.0308	BIRC3,SLC25A4
Estrogen Receptor Signaling	1.46	0.0122	NDUFA4,NDUFB3,PRKD3,SRC,VEGFA
Mitotic Roles of Polo-Like Kinase	1.45	0.0299	PTTG1,SLK
Role of JAK1 and JAK3 in γc Cytokine Signaling	1.43	0.029	CRLF2,IL7R
Ribonucleotide Reductase Signaling Pathway	1.4	0.0176	NFYA,SRC,VEGFA
Granzyme A Signaling	1.36	0.0267	NDUFA4,NDUFB3
Macropinocytosis Signaling	1.35	0.0263	PRKD3,SRC
Toll-like Receptor Signaling	1.33	0.0256	IL12B,TAB2
IL-7 Signaling Pathway	1.33	0.0256	IL7R,SLC2A1
Renal Cell Carcinoma Signaling	1.32	0.0253	SLC2A1,VEGFA
IL-17 Signaling	1.3	0.016	IL12B,TAB2,VEGFA

.

Supplementary Table S5. Primers used in real time PCR

Target (Gene fragment)	Sequence (5′-3′)	Sequence (3′-5′)	Ac no.
hTNFα	ATCTTCTCGAACCCCGAGTG	ATGAGGTACAGGCCCTCTGAT	NM_000594.4
hIL-6	CCTTCCAAAGATGGCTGAAA	CAGGGGTGGTTATTGCATCT	NM_000600.5
hIL-1β	CTGTACCTGTCCTGCGTGTT	AGACGGGCATGTTTTCTGCT	NM_000576.3
hIL-8	AGTCCTTGTTCCACTGTGCC	GTGCTTCCACATGTCCTCAC	NM_000584.4
hCPTIA	TGAGCGACTGGTGGGAGGAG	GAGCCAGACCTTGAAGTAGCG	NM_001876 [1]
hCD300A	CCTGCACAACAGTGACCAAC	CTGATGGCAACAGAGGGAT	NM_007261.4 [2]
hFNIP1	TTTGTTCCTCCCCACTGCTTCCCA	AGTAGCAGCAGCTCATTCCTTGGG	NM_133372.3 [3]
hIL-23A	AGCCGCCCGGGTCTT	TCCTTGAGCTGCTGCCTTTAG	NM_016584.3 [4]
hRPL9	CGGCGTAGTTTGGTGGAAAAA	CCACCATTTGTCAACCCGGA	NM_000661.5
hOLR1	AGCAAATTGTTCAGCTCCTTGTC	GCCCGAGGAAAATAGGTAACAGT	AB010710 [5]
hGAPDH	TCACCAGGGCTGCTTTTAAC	GACAAGCTTCCCGTTCTCAG	NM_002046.7

Supplementary Table S6. Composition of fatty acids mixture.

Fatty Acid		Catalog no.[1]	Composition (μl / 100 μl)
C 10:0	Capric acid	21409	1.49
C 12:0	Lauric acid	W261416	43.54
C 14:0	Myristic acid	M3128-10G	8.05
C 16:0	Palmitic acid	P5585	12.19
C 16:1	Palmitoleic acid	76169	1.80
C 18:0	Stearic acid	S4751	1.89
C 18:1	Oleic acid	O1383	9.95
C 18:2 n6	Linoleic acid	L1012	19.25
C 18:3 n3	Alpha Linolenic acid	L2376	1.84

[1]All FAs were purchased from Sigma- Aldrich, Chemical Co., USA, and were solubilized in ethanol prior to blending.

Supplementary Method S1. RNA Sequencing Protocol and Computational Pipeline

Library Construction and Sequencing. RNA-seq libraries were prepared at the Crown Genomics institute of the Nancy and Stephen Grand Israel National Center for Personalized Medicine, Weizmann Institute of Science. A bulk adaptation of the MARS-Seq protocol [6],[7] was used to generate RNA-Seq libraries for expression profiling of THP-1 treated cells. Replicates of high RNA integrity (RIN≥8) were processed. Briefly, 30 ng of input RNA from each sample was barcoded during reverse transcription and pooled. Following Agencourct Ampure XP beads cleanup (Beckman Coulter), the pooled samples underwent second strand synthesis and were linearly amplified by T7 in vitro transcription. The resulting RNA was fragmented and converted into a sequencing-ready library by tagging the samples with Illumina sequences during ligation, RT, and PCR. Libraries were quantified by Qubit and TapeStation as well as by qPCR for ActB housekeeping gene as previously described[6],[7]. Sequencing was done on an Illumina NovaSeq machine, using SP (100 cycles) protocol. The output was ~9.5 million single-end 100-bp reads per sample. Fastq files for each sample were generated by the usage bcl2fastq v2.20.0.422. **Sequence Data Analysis.** Poly-A/T stretches and Illumina adapters were trimmed from the reads using cutadapt[8]; resulting reads shorter than 30bp were discarded. Reads for each sample, were aligned independently to the Home sapiens reference genome, GRCh38_p13, using STAR (2.7.3a). The EndToEnd option was used and outFilterMismatchNoverLmax was set to 0.05. Deduplication was

carried out by flagging all reads that were mapped to the same gene and had the same UMI. Expression levels for each gene were quantified using htseq-count (version 0.11.2)[9], using the gene annotations downloaded from Ensembl (release 106). Counting was performed on the TES (Transcript End Site) region of each gene. The TES region was defined as 100 bp downstream and 1000 bases upstream, from the end of the gene. Only uniquely mapped reads were used to determine the number of reads mapped into each gene (union mode). UMI counts were corrected for saturation by considering the expected number of unique elements when sampling without replacement. Differential analysis was performed using DESeq2 package (1.26.0) [10] with the betaPrior, cooksCutoff and independentFiltering parameters set to False. Raw P values were adjusted for multiple testing using the procedure of Benjamini and Hochberg. Pipeline was run using snakemake[11]. Differentially expressed genes (DEGs), were determined by a p-adj of < 0.01 and absolute fold changes > 1.6 and max raw counts > 10. **Bioinformatics Analysis.** PCA and Hierarchical clustering (distance: Pearson's dissimilarity, method: Ward.d) was performed, based on the 1000 most variable genes. Unsupervised analysis was executed in order to explore a pattern of gene expression by clustering the genes based on genes that were determined as differential expressed (DE) genes as described above. K-Means clustering was performed. Standardized, log 2 normalized counts were used for the clustering analysis. Clustering analysis was performed with Rstudio v3.6.1. DEGs, heatmaps, canonical pathways and graphical networks were analyzed using Ingenuity Pathways Analysis (Ingenuity® Systems, www.ingenuity.com) to determine most significantly relevant biological functions and pathways.

Supplementary Method S2. Eicosanoids and Oxylipins Analysis.

Quantitative analysis of all metabolites was carried out by an external authorized laboratory, Creative Proteomics (45-1 Ramsey Road, Shirley, NY 11967, USA). MBSFL samples (Entoprotech Ltd., Israel; lot no. ME-290922) were analyzed in an LC-MS platform consisted of a Dionex Ultimate 3000 HPLC coupled to a Thermo LTQ-Orbitrap XL mass spectrometer. The LC system included quaternary pumps, a vacuum degassing system, thermostated autosampler, and a column oven. The HPLC column was a Phenomenex 2.0 mmx150 mm Synergi HydroRP-C18 (4 μ, 80 Å pore size) equipped with a guard cartidge of the same column chemistry. The LC gradient was adapted from Watrous et al., [12]. Solvent A was 70: 30 water: acetonitrile (v:v) containing 0.1% acetic acid. Solvent B was 50: 50 isopropanol: acetonitrile containing 0.02% acetic acid. Solvent C was isopropanol containing 0.02% acetic acid. The flow rate was 200 μl/ min and the column oven was

held at 45˚C. The autosampler was held at 4˚C. 10 µl of each sample was injected. The gradient conditions used were: Time 0-2 min, 1% solvent B and 0% solvent C. Column eluant was diverted to waste using a 2-position 6 port valve. At time= 2.0 min, Solvent B was increased to 50% with 0% solvent C, and a linear gradient from 50% to 65% B was run between 2.0 and 10 min while solvent C remained 0%. Solvent B then increased linearly to 87% B between 10 and 14 min with solvent C at 0%. Solvent C then was increased from 87% to 99% from 14-16 min while solvent B was dropped to 0%. Solvent C was then held constant at 99% until 24 min. Solvent C was then returned to 0% and solvent B was returned to 1% to re-equilibrate the column for 5 min. Column eluent was introduced to a Thermo LTQ-Orbitrap Velos mass spectrometer via a heated electrospray ionization source. The mass spectrometer was operated in negative ion mode at 60,000 resolution with full scan MS data collected from 200-700 m/z. Data-dependant product ion spectra were collected on the 4 most abundant ions at 7,500 resolution using the FT analyzer. The electrospray ionization source was maintained at a spray voltage of 4.5kV with sheath gas at 30 (arbitrary units) and auxillary gas at 10 (arbitrary units). The inlet of the mass spectromter was held at 350˚C, and the S-lens was set to 35%. The heated ESI source was maintained at 350˚C. Chromatographic alignment, isotope correction, peak identification and peak area calculations were performed using MAVEN software. Concentrations of each analyte were determined against the peak area of the internal standard. Confirmed analytes were identified by comparison of aligned LC-MS peaks to the median m/z values, retention times (RT), and MS/MS fragmentation spectra of authentic reference standards.

Supplementary Method S3. Mevalonate Pathway and Isoprenoids/ Cholesterol Analysis.

Quantitative analysis of all metabolites was carried out by an external authorized laboratory, Creative Proteomics (45-1 Ramsey Road, Shirley, NY 11967, USA). MBSFL samples (Entoprotech Ltd., Israel; lot no. ME-290922) were incubated at 45˚C to liquefy the oil and then aliquoted in 20 mg per tube. 500 µL of an internal standard solution (IS) of HMG-CoA-d10 was added. The samples were vortexed for 2 min, followed by centrifugation at 21,000 g for 10 min. The supernatant of each sample was transferred to an LC injection vial and dried under a nitrogen gas flow. The residues were reconstituted in 100 µL of 10% acetonitrile. After centrifugation, 10 µL aliquots of the sample solutions, and serially diluted standard solutions containing the IS were injected to run UPLCMRM/MS on a Water Acquity UPLC system coupled to a Sciex QTRAP 6500 Plus mass spectrometer operated in negative-ion mode. A C18 column (2.1*100 mm,

1.7 µm) and a mobile phase of tributylamine buffer (solvent A) and acetonitrile (solvent B) were used for gradient elution (10% to 80% B over 15 min) at 0.3 mL/min and 40 °C. Concentrations of the detected compounds were calculated by interpolating the constructed linear-regression calibration curves of individual compounds with the peak area ratios measured from sample solutions. Note: IPPP and DMAPP were not separated by LC or by MRM/MS and concentrations of the total of these two compounds in each sample was reported. 20 mg of each sample was dissolved in 400 µL of an internal solution (IS) of CoQ10-d9 in chloroform-methanol (1:1). A solution of standard substances of squalene, 2,3-oxidosqualene, MK-4, CoQ 6, 8, 9 and 10, and a mixture of dolichols 13-21 was prepared in IS solution and then serially diluted to have 9-point calibration solutions. 10 µL of each sample solution or each calibration solution was injected to run LC-MRM/MS on a Water Acquity UPLC system coupled to a Sciex QTRAP 6500 Plus mass spectrometer with positive-ion detection. A C8 column (2*100 mm, 2.5 µm) and a mobile phase of 0.1% formic acid in water (A) and 0.1% formic acid in isopropanol-acetonitrile (2:1) (B) was used for binary-solvent gradient elution (50% to 100% B over 18 min) at 0.35 mL/min and 55 °C. Concentrations of the detected compounds were calculated by interpolating the constructed linear-regression calibration curves with peak area or peak area ratios measured from the sample solutions.

References

1. Kohjima, M.; Enjoji, M.; Higuchi, N.; Kato, M.; Kotoh, K.; Yoshimoto, T.; Fujino, T.; Yada, M.; Yada, R.; Harada, N.; et al. Re-Evaluation of Fatty Acid Metabolism-Related Gene Expression in Nonalcoholic Fatty Liver Disease. *Int. J. Mol. Med.* **2007**, *20*, 351–358, doi:10.3892/ijmm.20.3.351.
2. Ju, X.; Zenke, M.; Hart, D.N.J.; Clark, G.J. CD300a/c Regulate Type i Interferon and TNF-α Secretion by Human Plasmacytoid Dendritic Cells Stimulated with TLR7 and TLR9 Ligands. *Blood* **2008**, *112*, 1184–1194, doi:10.1182/blood-2007-12-127951.
3. Hasumi, H.; Baba, M.; Hong, S.B.; Hasumi, Y.; Huang, Y.; Yao, M.; Valera, V.A.; Linehan, W.M.; Schmidt, L.S. Identification and Characterization of a Novel Folliculin-Interacting Protein FNIP2. *Gene* **2008**, *415*, 60–67, doi:10.1016/j.gene.2008.02.022.
4. Hor, Y.T.; Voon, D.C.C.; Koo, J.K.W.; Wang, H.; Lau, W.M.; Ashktorab, H.; Chan, S.L.; Ito, Y. A Role for RUNX3 in Inflammation-Induced Expression of IL23A in Gastric Epithelial Cells. *Cell Rep.* **2014**, *8*, 50–58, doi:10.1016/j.celrep.2014.06.003.
5. Yamagata, K.; Tusruta, C.; Ohtuski, A.; Tagami, M. Docosahexaenoic Acid Decreases TNF-α-Induced Lectin-like Oxidized Low-Density Lipoprotein Receptor-1 Expression in THP-1 Cells. *Prostaglandins Leukot. Essent. Fat. Acids* **2014**, *90*, 125–132, doi:10.1016/j.plefa.2013.12.011.
6. Diego Adhemar Jaitin; Ephraim Kenigsberg; Hadas Keren-Shaul; Naama Elefant; Franziska Paul; Irina Zaretsky; Alexander Mildner; Nadav Cohen; Steffen Jung; Amos Tanay; et al. Massively Parallel Single-Cell RNA-Seq for Marker-Free Decomposition of Tissues into Cell Types. *Science (80-.).* **2014**, *343*, 772–776, doi:10.1126/science.1247651.
7. Keren-Shaul, H.; Kenigsberg, E.; Jaitin, D.A.; David, E.; Paul, F.; Tanay, A.; Amit, I. MARS-Seq2.0: An Experimental and Analytical Pipeline for Indexed Sorting Combined with Single-Cell RNA Sequencing. *Nat. Protoc.* **2019**, *14*, 1841–1862, doi:10.1038/s41596-019-0164-4.
8. Martin, M. Cutadapt Removes Adapter Sequences from High-Throughput Sequencing Reads. *EMBnet.journal* **2011**, *17*, 10–12, doi:https://doi.org/10.14806/ej.17.1.200.
9. Anders, S.; Pyl, P.T.; Huber, W. HTSeq-A Python Framework to Work with High-Throughput Sequencing Data. *Bioinformatics* **2015**, *31*, 166–169, doi:10.1093/bioinformatics/btu638.
10. Love, M.I.; Huber, W.; Anders, S. Moderated Estimation of Fold Change and Dispersion

for RNA-Seq Data with DESeq2. *Genome Biol.* **2014**, *15*, 1–21, doi:10.1186/s13059-014-0550-8.

11. Köster, J.; Rahmann, S. Snakemake- a Scalable Bioinformatics Workflow Engine. *Bioinformatics* **2012**, *28*, 2520–2522, doi:10.1093/bioinformatics/bts480.
12. Watrous, J.D.; Niiranen, T.J.; Lagerborg, K.A.; Henglin, M.; Xu, Y.J.; Rong, J.; Sharma, S.; Vasan, R.S.; Larson, M.G.; Armando, A.; et al. Directed Non-Targeted Mass Spectrometry and Chemical Networking for Discovery of Eicosanoids and Related Oxylipins. *Cell Chem. Biol.* **2019**, *26*, 433-442.e4, doi:10.1016/j.chembiol.2018.11.015.
